# Crystal Structures and Phase Stability of the Li_2_S–P_2_S_5_ System from First Principles

**DOI:** 10.1021/acs.chemmater.3c01793

**Published:** 2023-10-23

**Authors:** Ronald L. Kam, KyuJung Jun, Luis Barroso-Luque, Julia H. Yang, Fengyu Xie, Gerbrand Ceder

**Affiliations:** † Materials Science Division, Lawrence Berkeley National Laboratory, Berkeley, California 94720, United States; ‡ Department of Materials Science and Engineering, University of California, Berkeley, California 94720, United States

## Abstract

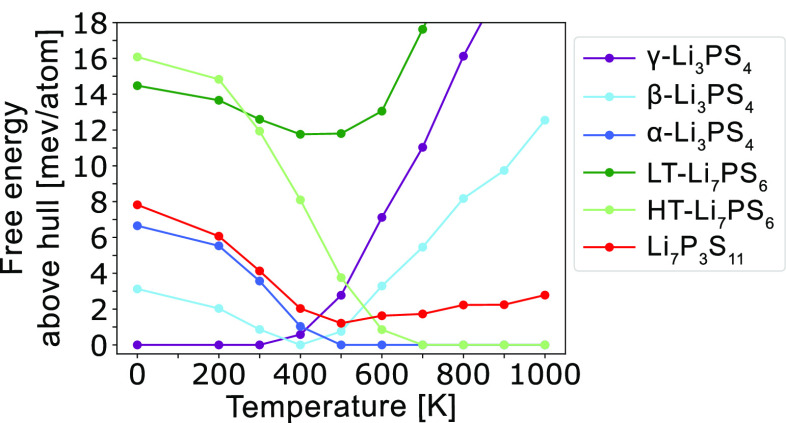

The Li_2_S–P_2_S_5_ pseudo-binary system has been a valuable source of promising superionic conductors, with α-Li_3_PS_4_, β-Li_3_PS_4_, HT-Li_7_PS_6_, and Li_7_P_3_S_11_ having excellent room-temperature Li-ion conductivity >0.1 mS/cm. The metastability of these phases at ambient temperature motivates a study to quantify their thermodynamic accessibility. Through calculating the electronic, configurational, and vibrational sources of free energy from first principles, a phase diagram of the crystalline Li_2_S–P_2_S_5_ space is constructed. New ground-state orderings are proposed for α-Li_3_PS_4_, HT-Li_7_PS_6_, LT-Li_7_PS_6_, and Li_7_P_3_S_11_. Well-established phase stability trends from experiments are recovered, such as polymorphic phase transitions in Li_7_PS_6_ and Li_3_PS_4_, and the instability of Li_7_P_3_S_11_ at high temperature. At ambient temperature, it is predicted that all superionic conductors in this space are indeed metastable but thermodynamically accessible. Vibrational and configurational sources of entropy are shown to be essential toward describing the stability of superionic conductors. New details of the Li sublattices are revealed and are found to be crucial toward accurately predicting configurational entropy. All superionic conductors contain significant configurational entropy, which suggests an inherent correlation between fast Li diffusion and thermodynamic stability arising from the configurational disorder.

## Introduction

**Figure 1:**
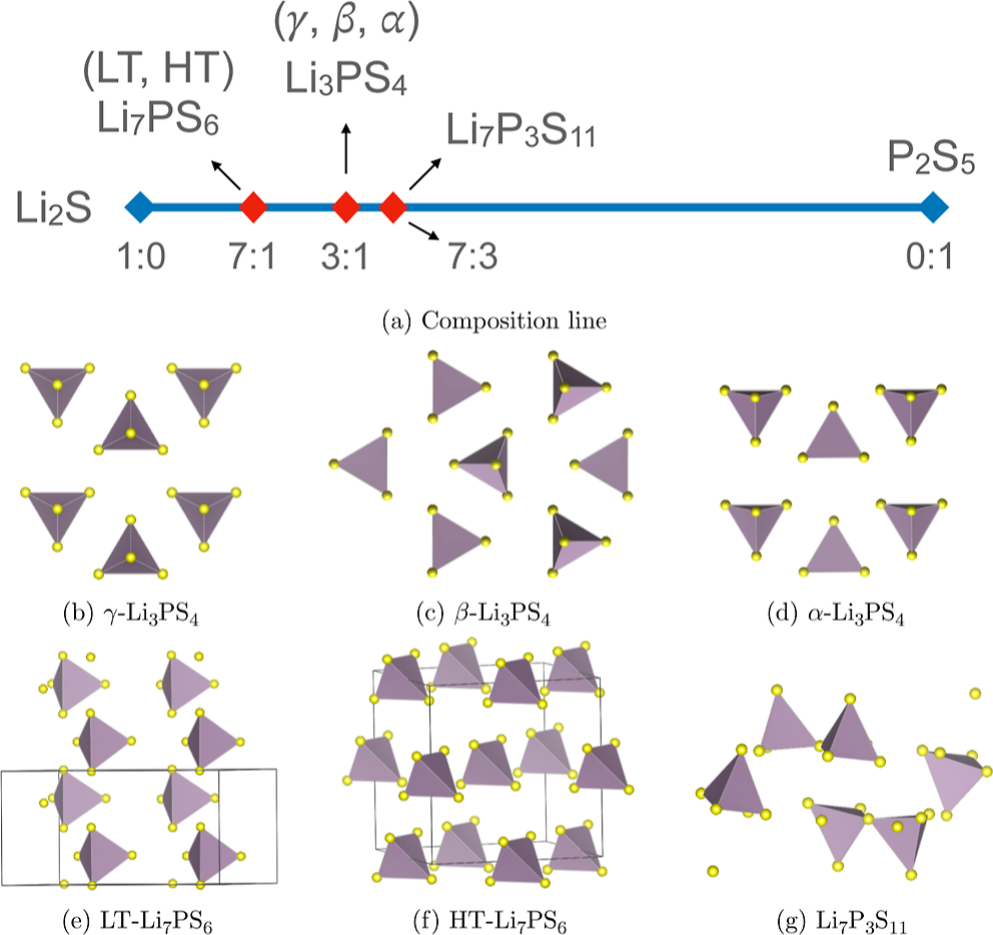
Composition line and arrangements of PS_4_ tetrahedral units in the Li_2_S–P_2_S_5_ system. (a) Location of Li_7_PS_6_, Li_3_PS_4_, and Li_7_P_3_S_11_ on the composition line, labeled by the ratios of Li_2_S to P_2_S_5_. Arrangements of PS_4_ and P_2_S_7_ units in (b) γ-Li_3_PS_4_, (c) β-Li_3_PS_4_, (d) α-Li_3_PS_4_, (e) LT-Li_7_PS_6_, (f) HT-Li_7_PS_6_, and (g) Li_7_P_3_S_11_. Unit cell boxes are drawn for LT-Li_7_PS_6_ and HT-Li_7_PS_6_ to show their cubic and orthorhombic PS_4_ arrangement, respectively.

The global transition to sustainable energy sources necessitates the continued development of energy storage technologies that enable increased deployment of intermittent energy sources (i.e., wind and solar power) and electrification of transportation.^1^ Lithium (Li) all solid-state batteries (ASSBs) can significantly improve the safety and energy density compared to conventional Li-ion batteries using organic liquid electrolytes.^2−4^ Discovery and development of novel superionic conductors with Li-ion conductivity on the order of organic liquid electrolytes (>0.1 mS/cm) are crucial toward enabling ASSBs to have similar power densities as conventional Li-ion batteries.^4^ The pseudo-binary Li_2_S–P_2_S_5_ composition space has proven to be a particularly rich source of promising Li superionic conductors. Several crystalline compounds can be synthesized by combining Li_2_S and P_2_S_5_ precursors in varying ratios (Figure 1a),^5^ with the notable phases being the α, β, and γ polymorphs of Li_3_PS_4_, high-temperature (HT) and low-temperature (LT)-Li_7_PS_6_, and Li_7_P_3_S_11_. Among these, α-Li_3_PS_4_, β-Li_3_PS_4_, HT-Li_7_PS_6_, and Li_7_P_3_S_11_ are superionic conductors.^6,7^ Although amorphous phases with these compositions also exist,^8−10^ the focus of our study will be on understanding the relative phase stability of the crystalline phases only.

The crystalline phases in the Li_2_S–P_2_S_5_ space are composed of periodically arranged PS_4_ tetrahedra, which are either isolated or form P_2_S_7_ ditetrahedra. Li atoms are located between these units and coordinated by S atoms. The Li_7_PS_6_ polymorphs also contain free S atoms that are only coordinated with Li atoms. Different phases can be identified by their distinct orientations of PS_4_ and P_2_S_7_ groups, which are shown in Figure 1. The Li_3_PS_4_ and Li_7_PS_6_ polymorphs are all composed of isolated PS_4_ groups. In γ-Li_3_PS_4_, these PS_4_ groups are unidirectional, with all apexes facing the same direction (apexes face out of the page in Figure 1b). In β-Li_3_PS_4_, PS_4_ groups are arranged in alternating zigzag chains, with each chain containing apexes that face the same direction, while apexes in the adjacent chain face the opposite direction (Figure 1c).^11^ The α-Li_3_PS_4_ polymorph also contains PS_4_ with oppositely facing apexes, but these are arranged in alternating columns (Figure 1d). In both Li_7_PS_6_ polymorphs, all PS_4_ groups face the same direction but differ in their spatial distributions.^12^ In LT-Li_7_PS_6_, PS_4_ is arranged with orthorhombic symmetry (Figure 1e), while in HT-Li_7_PS_6_, the PS_4_ is arranged with face-centered cubic symmetry (Figure 1f).^12^ Li_7_P_3_S_11_ is composed of both P_2_S_7_ and PS_4_ units (Figure 1g).^13^

According to previous experimental and computational studies, the superionic conductor phases are all metastable at ambient temperature.^5,10^ Among the Li_3_PS_4_ polymorphs, γ is the stable phase at room temperature but has low Li conductivity, while β and α are the high-temperature fast-conducting phases.^11,14^ β-Li_3_PS_4_ has been stabilized at room temperature as nanoporous particles from solution-state synthesis.^15,16^ This phase has also been stabilized through mechanochemical synthesis involving ball milling to form an amorphous phase and a subsequent heat treatment to recrystallize.^9^ An analogous Si-doped Li_3.25_Si_0.25_P_3.75_S_4_ structure, where Si substitutes into phosphorus (P) sites, has also been stabilized at room temperature.^17^ α-Li_3_PS_4_ has recently also been stabilized at room temperature via a rapid heating and quenching technique.^18^ This discovery indicates that the energy differences among the three Li_3_PS_4_ polymorphs at room temperature should be small, allowing for the metastable α and β to be thermodynamically accessible at ambient temperature. HT-Li_7_PS_6_ is only stable at elevated temperatures (*T* > 483 K)^12^ but has been successfully stabilized at room temperature through halide atom substitution into S sites, typically to form the Li_6_PS_5_X composition (X = Cl, Br, or I).^19−22^ Synthesis of Li_7_P_3_S_11_ usually requires ball-milling to its amorphous form before recrystallization above its glass transition temperature of around 500 K.^5,13,23^ Heat treatment at higher temperatures (*T* > 800 K) is not possible, as Li_7_P_3_S_11_ phase separates to Li_4_P_2_S_6_ and Li_3_PS_4_.^23^

The metastable nature of these superionic conductors motivates our first-principles study with the objective to understand their thermodynamic accessibility at finite temperature, rationalize experimental trends, and potentially propose new synthesis procedures. To model the free energy of each phase, we consider contributions from the electronic structure, configurational disorder, and vibrational modes. We find that including both configurational and vibrational entropy is necessary to correctly predict free energies, in agreement with a previous study on the Li_1+2*x*_Zn_1+*x*_PS_4_ system.^24^

We model configurational Li-vacancy disorder with well-established lattice model methods,^25,26^ which have been previously used to study a range of alkali-ion intercalation cathodes and solid electrolytes.^27−29^ To properly model the configurational disorder in HT-Li_7_PS_6_, α-Li_3_PS_4_, β-Li_3_PS_4_, and Li_7_P_3_S_11_, we require accurate structural models to define the set of distinct sites that Li can occupy, which we term the Li sublattice. There are conflicting reports about the specific sites that make up the Li sublattices arising from different characterization techniques. More specifically, in α-Li_3_PS_4_, β-Li_3_PS_4_, and HT-Li_7_PS_6_, neutron diffraction (ND) refinements^14,19^ have identified more Li sites and increased site disorder as compared to X-ray diffraction (XRD) refinements.^11,12,30^ In Li_7_P_3_S_11_, XRD and ND identify fully ordered, but entirely different Li sublattices.^13,31^ A more recent ab initio molecular dynamics (AIMD) study proposing 15 potential Li sites in Li_7_P_3_S_11_ introduces uncertainty to the exact state of Li order, since these new sites can in principle be partially occupied.^32^ Because of these conflicting reports, we dedicate a large portion of this study toward clarifying the Li arrangement in these structures, the details of which we find to be essential for recovering experimental thermodynamic trends.

For each disordered phase, we assess the validity of various proposed Li sublattices primarily by analyzing atomic relaxation distances and comparing Li site disordering behavior to experimental reports. Upon obtaining the most representative Li sublattice, we train a cluster expansion (CE), which can rapidly evaluate total energies of any Li-vacancy configuration within the given Li sublattice.^25^ Using the CE, we perform Monte Carlo (MC) sampling to determine the configurational entropy, free energy, and Li site disordering behavior as a function of temperature.

The CE formally represents the energy of a disordered crystal structure as a summation over contributions from local, multisite (cluster) configurations and their associated interaction energies.^25,33^ The expression for CE energy is shown in eq 1, where σ⃗ is the vector encoding the species occupying each lattice site, β is the index for a symmetrically distinct cluster, *J*_β_ is the effective cluster interaction (ECI) energy, and ⟨Φ(σ)⟩_β_ is the correlation function describing the crystal-averaged cluster configuration. The ECI are determined from regularized linear regression techniques, using a training set of distinct DFT-relaxed configurations and energies.^25^ Regularization hyperparameters were chosen to minimize the 5-fold cross-validation (CV) root-mean-square error (RMSE).
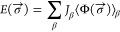
1Monte Carlo (MC) sampling is then performed using the CE in the canonical ensemble to predict Li site disorder, identify new ground-state (lowest energy) structures, and calculate configurational thermodynamic properties through thermodynamic integration (more in Methods section).

Vibrational free energy contributions are captured in the ground state of each phase, by performing harmonic phonon calculations.^34^ By incorporating the electronic, vibrational, and configurational free energy contributions, we assess the thermodynamic stability in the Li_2_S–P_2_S_5_ phase space, recovering well-established experimental observations.

This paper is organized as follows. (1) We first present the pseudo-binary Li_2_S–P_2_S_5_ phase diagram. The thermodynamic stability of each phase at finite temperature is evaluated, and potential synthesis procedures for metastable phases are proposed. (2) For each composition, we discuss the appropriate choice of refined structure for each polymorph by comparing the validity of previously proposed models. Phase stability trends between polymorphs are examined in detail, with a focus on identifying phase transitions and quantifying the contributions of vibrational and configurational entropy toward stability. (3) In the Discussion section, we draw further connections to previously proposed experimental synthesis strategies and explore a potential correlation between superionic conductivity and high configurational entropy.

## Results

### Phase Stability in the Li_2_S–P_2_S_5_ System

**Figure 2:**
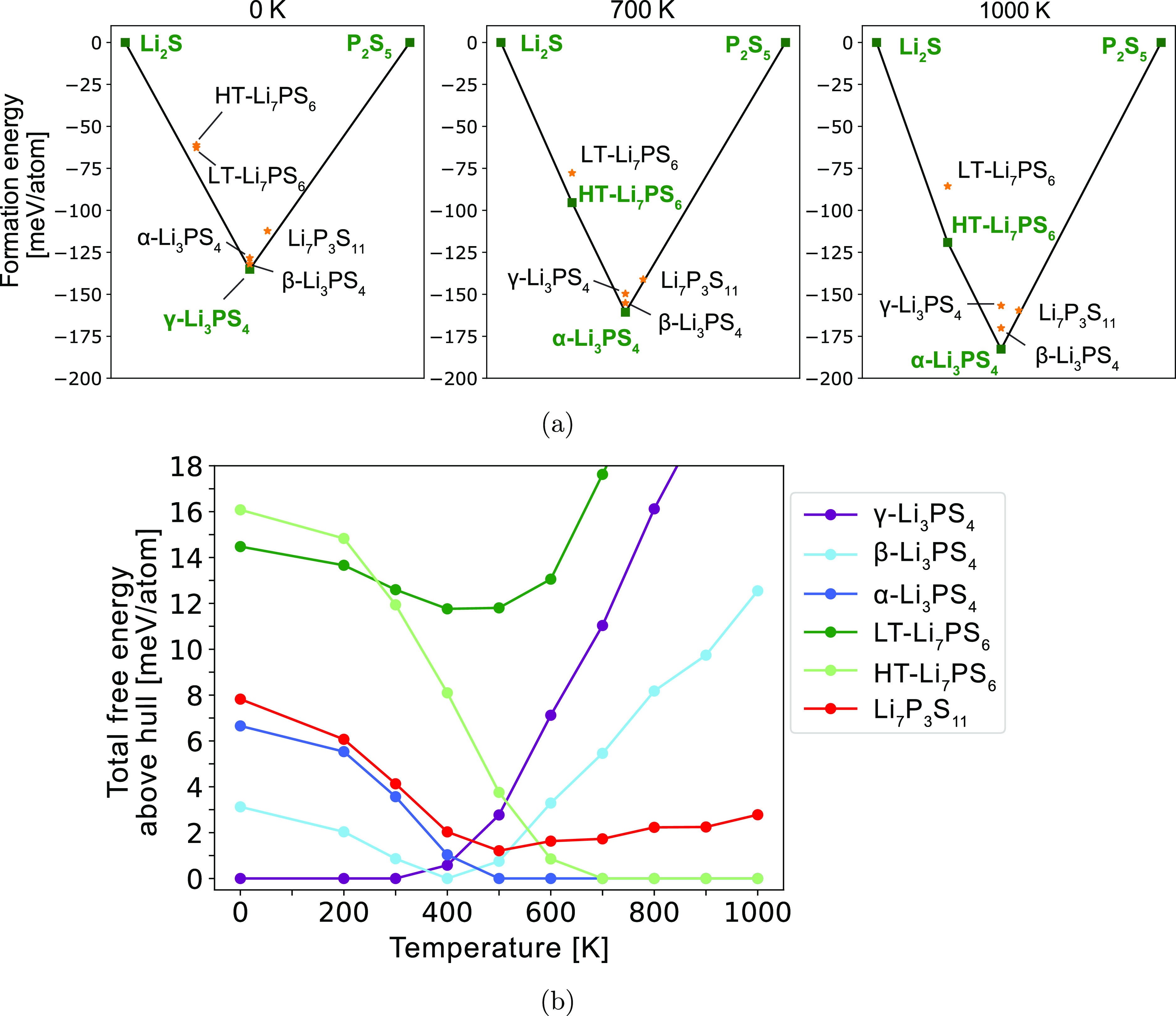
Phase stability of the Li_2_S–P_2_S_5_ pseudo-binary system. (a) Phase diagram at 0, 700, and 1000 K. Solid lines denote the convex hull. Stable phases that are on the convex hull are marked with squares and are labeled in green. Unstable phases are marked with gold stars and are labeled in black. (b) Free energy above the hull for all phases from 0 to 1000 K.

The pseudo-binary Li_2_S–P_2_S_5_ phase diagram is presented in Figure 2a, and the energies above the hull (*E*_hull_) as a function of temperature are shown in Figure 2b. The convex hull is a typical construction to obtain stable phases and represents the collection of thermodynamic ground states into which all other phases have a driving force to convert.^35^ All computed formation free energies used to construct the phase diagram are shown in Figure S7. At 0 K, the only stable phases on the convex hull are γ-Li_3_PS_4_ and the end points, Li_2_S and P_2_S_5_ (Figure 2a). At 700 K, HT-Li_7_PS_6_ is stabilized and appears on the hull. Since reported synthesis procedures for LT and HT-Li_7_PS_6_ typically do not require mechanical milling or quenching,^12,20^ it may be surprising that they are unstable at 300 K—13 and 12 meV/atom above the hull, respectively (Figure 2b). It is likely that the thermodynamically favored phase separation of HT-Li_7_PS_6_ to Li_2_S and Li_3_PS_4_ is kinetically hindered at room temperature. Instead, HT-Li_7_PS_6_ is found to transform to LT-Li_7_PS_6_ upon cooling, a potentially more facile process as it merely involves shifting the PS_4_ locations (Figure 1). Thus, an appropriate solid-state synthesis procedure would be to perform sufficiently high-temperature (*T* > 600 K) synthesis to stabilize HT-Li_7_PS_6_, before a relatively rapid cooling process to bypass the phase separation to Li_2_S and Li_3_PS_4_.

For the Li_3_PS_4_ composition, our calculations in Figure 2b predict phase transformations from γ → β → α with an increasing temperature, which is consistent with experiments. Since β-Li_3_PS_4_ is less than 1 meV/atom above the hull at 300 K (Figure 2b), it is plausible that nanoporous synthesis and mechanical milling techniques can lead to its stabilization at room temperature.^9,15^ The α-Li_3_PS_4_ polymorph is only slightly less stable than β at 300 K (*E*_hull_ = 4 meV/atom), which explains why α can also be stabilized at ambient temperature through a rapid heating and quenching procedure.^18^ Rapid heating of the Li_3_PS_4_ glass to temperatures in the stability range of β enables nucleation of metastable α particles that are only slightly less stable than β, which is possible by the Ostwald step rule.^18,36^ Rapid quenching can then obstruct the commonly observed direct transition from α to γ,^11,14^ which is possible as their energy difference is only 4 meV/atom at 300 K.

Li_7_P_3_S_11_ (red curve in Figure 2b) is metastable across all temperatures as its energy is never low enough to be on the convex hull, which agrees with prior experimental studies.^13,23^ At 300 K, it is 4 meV/atom above the convex hull. As the temperature increases to 500 K, its *E*_hull_ decreases to a minimum of 1.4 meV/atom. Further increases in temperature lead to greater *E*_hull_. Thus, an ideal synthesis temperature should be around 500 K, corresponding to a minimum *E*_hull_. This temperature is remarkably close to its experimentally observed glass transition temperature and helps rationalize why heat treatments near this temperature have been successful for recrystallization.^7,23^ The increasing instability with respect to temperature helps explain the experimentally observed tendency to phase-separate to Li_3_PS_4_ and Li_4_P_2_S_6_ at temperatures greater than 800 K.^9^ The source of this instability is the competition with α-Li_3_PS_4_, its neighboring stable point, which lowers its free energy more with increasing temperature, therefore increasing the convex hull depth (Figure 2a). We will show in the next section that this arises from the high configurational entropy in α-Li_3_PS_4_.

### Li_3_PS_4_ Polymorphs

γ-Li_3_PS_4_ (*Pnm*2_1_) is the stable polymorph at room temperature and has a very low Li conductivity of 3(10^–4^) mS/cm.^11^ The reported XRD and ND refinements are in excellent agreement with each other, showing an ordered Li sublattice comprising fully occupied Li1 (4b) and Li2 (2a) sites. Since there is no ambiguity in these refinements, we use this structure to model γ-Li_3_PS_4_.^11,14^

### β-Li_3_PS_4_ Structure

Upon heating, γ transforms to β-Li_3_PS_4_ at around 575 K, crystallizing in the orthorhombic *Pnma* space group, which leads to a lattice volume expansion by ∼3%.^11^ The zigzag ordering of PS_4_ units generates a different Li sublattice with more sites than in γ, leading to the potential for disorder. At around 600 K, XRD refinements have reported Li atoms occupying Li1’ (8d), Li2’ (4b), and Li3’ (4c) sites, with fractional occupancies of 1, 0.7, and 0.3, respectively (XRD refined sites are labeled with apostrophes and ND refined sites without apostrophes for clarity in this discussion).^11^ A more recent ND refinement proposes a slightly different model, with reported site splitting of Li1’ (8d) to Li1A (8d) and Li1B (8d), and splitting of Li2’ (4b) to Li2 (8d), while retaining its *Pnma* symmetry.^14^ The four distinct Li sites refined by ND are all partially occupied.

**Figure 3:**
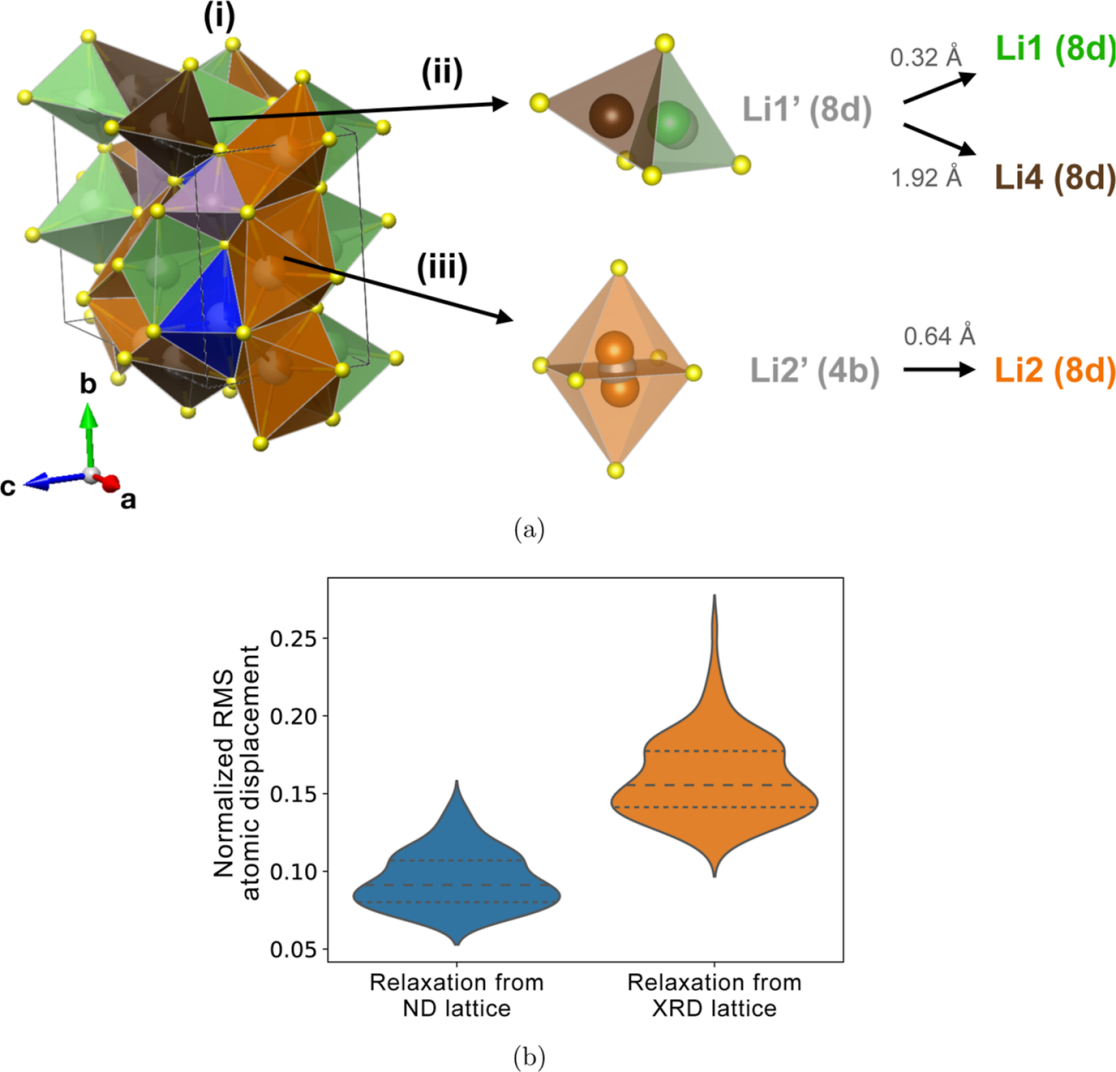
Structure of β-Li_3_PS_4_. (a) (i) Unit cell with Li1 (8d) (green), Li2 (8d) (orange), Li3 (4c) (blue), and Li 4 (8d) (brown) sites. (ii) Splitting of Li1’ (8d) (gray) to Li1 (8d) and Li4 (8d). (iii) Splitting of Li2’ (4b) (gray) from square planar coordination to 5-fold coordinated Li2 (8d). (b) Distributions of atomic relaxations in DFT starting from ideal ND and XRD refined structures. The extent of atomic relaxations is measured in NRMS atomic displacements. The dashed lines denote the 25, 50, and 75 percentiles in the distributions.

We analyze the geometric discrepancy between the XRD and ND refinements of β-Li_3_PS_4_ by inspecting the Li coordination environments in the XRD and ND sites. In Figure 3a, we show (i) the unit cell, (ii) splitting of Li1’ (8d), and (iii) splitting of Li2’ (4b). The splitting of the Li1’ (8d) site [gray in Figure 3a(ii)] in fact yields two distinct sites: Li1A (8d) and Li1B (8d) [green and brown in Figure 3a(ii), respectively]. Li1A (8d) is essentially identical to Li1’ (8d), while Li1B (8d) is its face-sharing neighbor 1.7 Å away. The emergence of Li1B as a new Li site can be detected by ND, while in XRD it has not been detected, likely due to the small X-ray scattering factor of Li. Since Li1A and Li1B sites are not related to each other, we will refer to Li1A as Li1 (8d) and Li1B as Li4 (8d) in the following discussion. Li2’ (4b) [gray in Figure 3a(iii)], with square planar coordination, splits into two neighboring and face-sharing Li2 (8d) sites [orange in Figure 3a(iii)], each with 5-fold coordination. XRD was unable to distinguish the two neighboring Li2 (8d) sites, which are only 1.3 Å apart, and instead identified just one Li2’ (4b) site.

To assess the accuracy of XRD and ND refinements of β-Li_3_PS_4_, we examine for all atomic positions in the DFT-relaxed configurations the deviation from their XRD and ND refined sites. This is measured by calculating the normalized root-mean-square (NRMS) displacement of relaxed atomic locations from the ND and XRD-refined β-Li_3_PS_4_ lattices. The atoms of a relaxed structure are mapped back to a refined lattice site to construct the “refined” structure. The atoms of the relaxed and refined structures are then placed on an averaged lattice (in Cartesian coordinates) that minimizes the NRMS displacement, which is defined in eq 2, where Δ*x*_*i*_ is the displacement of atom i between the DFT-relaxed structure and the ND or XRD-refined model in Cartesian coordinates, *N* is the number of atoms, and *V* is the cell volume. The procedure described is performed using the StructureMatcher module in the Pymatgen python package.^37^
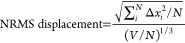
2In Figure 3b, we show violin plots of the distributions of NRMS displacement from the ND-refined structure (blue) and the XRD-refined structure (orange). NRMS displacement from the ND structure is significantly smaller compared to the XRD structure since the third quartile of the ND and the second quartile of the XRD distributions do not overlap (Figure 3b). The distribution of relaxations from the ND structure also has a smaller range and thus less probable outliers, suggesting that the ND refinement is more accurate.

**Figure 4:**
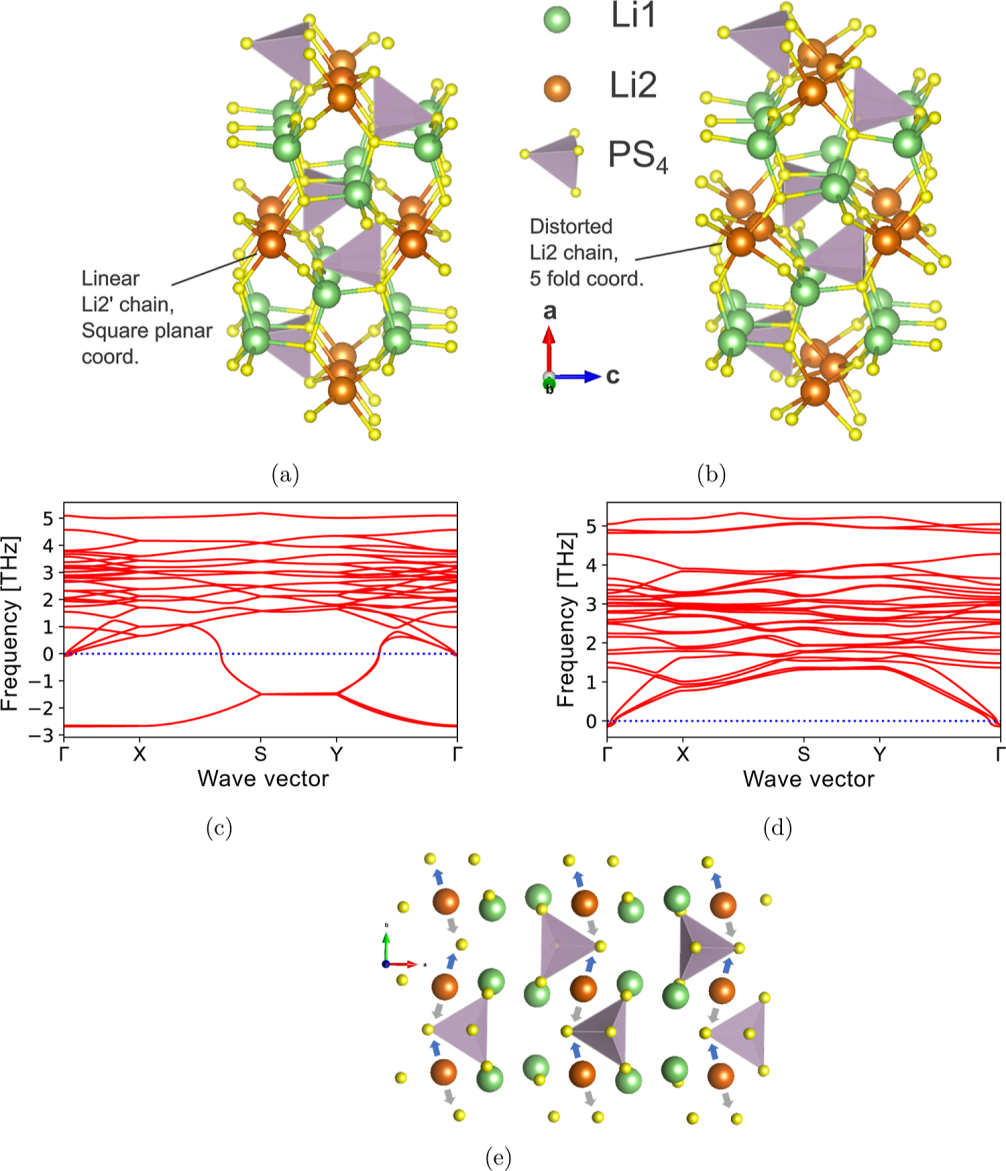
Structures of the (a) XRD ground state (XRD-GS) and (b) ND ground state (ND-GS) of β-Li_3_PS_4_. XRD-GS contains linear ordering of Li1 and Li2 atoms along [010] and [001], while ND-GS has distorted, staggered chains along [010]. Phonon dispersions of the (c) XRD-GS and (d) ND-GS. (e) Visualizing an imaginary optical mode of XRD-GS at Γ, showing collective motion of Li2 atoms (orange). The blue and gray arrows indicate mode displacement directions.

To gain insight into the physical nature of ND- and XRD-refined sites in β-Li_3_PS_4_, we examine two low-energy structures that were previously proposed as the ground state in separate first-principles studies.^38,39^ These highly similar structures are shown in Figure 4a,b. One contains fully occupied Li1’ (8d) and Li2’ (4b) sites, which yields well-ordered linear chains of Li1’ and Li2’ atoms along [010] and [001] and retains the *Pnma* symmetry of the underlying lattice—we will refer to this as the XRD ground state (XRD-GS) (Figure 4a). The other proposed structure is the true, lowest energy ground state in our data set, which is reported to have fully occupied Li1’ (8d) and Li2’ (4b) sites, but the square planar coordinated Li2’ atoms are displaced off-center to a neighboring 5-fold coordination environment, characteristic of the ND-refined Li2 (8d) site—we will refer to this as the ND ground state (ND-GS) (Figure 4b). The Li2 chain of atoms in ND-GS is staggered along [010], which leads to decreased symmetry (*P*2_1_2_1_2_1_) compared with XRD-GS (*Pnma*). The Li site fractional occupancies of ND-GS can be described on the basis of the ND-refined sites as *x*_Li1_ = 1, *x*_Li2_ = 0.5, and *x*_Li3_ = *x*_Li4_ = 0.

Although Li2’ (4b) is located merely 0.6 Å from a neighboring Li2’ (8d) site, the decrease in site energy is substantial, as ND-GS is 3.4 meV/atom lower than XRD-GS. Furthermore, when comparing phonon dispersion spectra, we find that XRD-GS is dynamically unstable with 2 nearly degenerate optical imaginary modes (Figure 4c), while ND-GS is dynamically stable with no imaginary modes (Figure 4d), agreeing well with previous reports.^38^ When visualizing the XRD-GS imaginary optical modes at the Γ wave vector, we observe a collective motion of Li2’ atoms (Figure 4e). This indicates that the XRD-refined Li2’ (4b) site is a high energy transition state for Li hopping between two neighboring Li2 (8d) sites. These findings highlight the importance of distinguishing fine details of the Li sublattice as substantial differences in physical behavior can arise when site locations are slightly perturbed.

**Figure 5:**
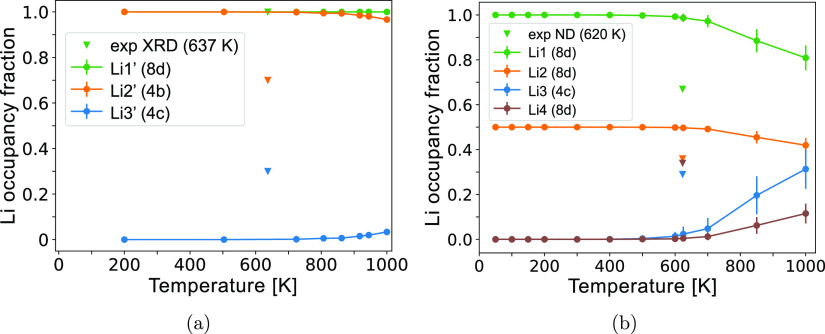
Li site fractional occupancies in β-Li_3_PS_4_ across temperature in MC simulations for the (a) XRD-refined lattice and (b) ND-refined lattice.

The thermodynamic disordering behavior of the XRD- and ND-refined structures at elevated temperature is also compared. We fit separate cluster expansions on each lattice and performed MC simulations to predict the Li site disorder as a function of temperature. In Figure 5, the Li site fractional occupancies across temperature are plotted. The XRD structure begins to disorder from XRD-GS at approximately 900 K, and by 1000 K, changes in the Li fractional occupancies are still relatively small, yielding poor agreement with the experimental XRD refinement (Figure 5a). The ND structure begins to disorder from ND-GS at a lower temperature of about 600 K, and by 1000 K, it has significant changes in its Li fractional occupancies, highlighted by Li1 (8d) and Li3 (4c) having occupancies of 0.8 and 0.3, respectively. These values show reasonable agreement with the ND refinement at 620 K (0.7 and 0.3) (triangles in Figure 5b). Our simulations of both the XRD and ND structures underestimate the experimentally reported configurational disorder. However, the ND structure is predicted to have greater disorder and thus better agreement with experiment, suggesting that the ND refinement is more accurate. Specifically, introducing the Li4 (8d) site and increasing multiplicity of Li2’ (4b) to Li2 (8d) generate more configurational states that appear essential toward accurately describing the thermodynamics of this phase.

### α-Li_3_PS_4_ Structure

**Figure 6:**
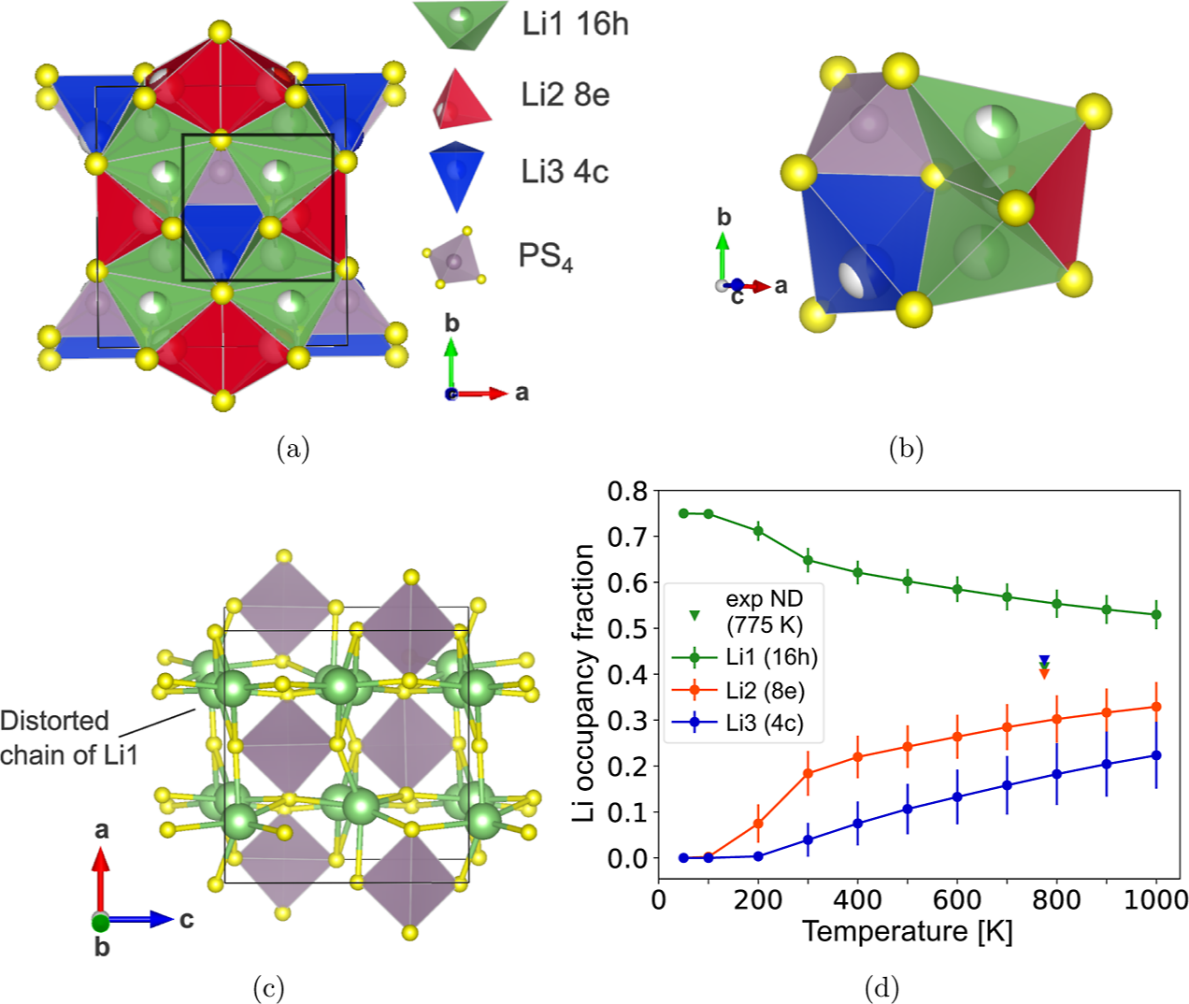
Structure of α-Li_3_PS_4_. (a) Unit cell with Li1 (16h) (green), Li2 (8e) (red), and Li3 (4c) (blue) sites. The boxed region is shown in greater detail in (b) to display the local Li coordination. Nearest Li1 and Li2 sites are face-sharing to form a connected Li channel along [010]. Two adjacent channels are connected by Li3 sites, which are edge-sharing with Li1. (c) Ground-state structure with monoclinic *P*2_1_/*c* symmetry. Li atoms occupy only Li1 sites, forming distorted linear chains of Li along [010] and [001]. (d) Li site fractional occupancies from MC simulations.

At high temperature (*T* > 725 K), β transforms to the orthorhombic *Cmcm* α-Li_3_PS_4_, increasing symmetry (*Cmcm* is a supergroup of *Pnma*) and slightly decreasing in density (1.6%).^14^ ND refinements report a Li sublattice containing Li1 (16h), Li2 (8e), and Li3 (4c) sites with high degree of disorder, as indicated by the isotropic Li fractional occupancies of around 0.4.^14^ An earlier refinement with XRD was deemed inconclusive, as only 1/3 Li atoms in the formula unit were refined to 1 distinct site, and there were large errors in the atomic displacement parameter (ADP).^30^ The ND refinement shows significant improvement by locating 2.9/3 Li and containing lower error in ADP.^14^ Therefore, we use the ND-refined structure, which contains 3 tetrahedral Li sites over which Li atoms can disorder, to construct our cluster expansion for α-Li_3_PS_4_. The disordered unit cell and local Li coordination of α-Li_3_PS_4_ are shown in Figure 6a,b, respectively. We can observe that α-Li_3_PS_4_ contains a well-connected 1D channel of face-sharing Li1–Li2–Li1 sites along [010] (Figure 6b), which can be associated with fast Li-ion conduction.^40^ The Li3 sites, which are edge-sharing with Li1, serve to bridge adjacent Li1–Li2–Li1 channels.

From MC simulated annealing, we find the ground state of α-Li_3_PS_4_ to be 3.2 meV/atom above the ground state of the β polymorph, and 8.0 meV/atom above the γ polymorph. The ground state of α-Li_3_PS_4_ is shown in Figure 6c. Li atoms only occupy the Li1 (16h) sites and form distorted linear Li chains along [010] and [001]. This indicates that Li1 (16h) sites are the most stable and their face-sharing Li2 (8e) neighbors are higher energy intermediate sites that facilitate rapid Li diffusion. Similarly, the Li3 (4c) sites are higher energy intermediate sites that connect adjacent Li1–Li2–Li1 channels and promote 3D conductivity.^14^ We note that this ground-state structure contains a small monoclinic distortion (*P*2_1_/*c*, lattice angle γ = 86.4°) arising from slightly rotated PS_4_ groups. We observe that many enumerated Li orderings of this structure contain similar symmetry-breaking lattice distortions after DFT relaxation, which indicates that the tilting of the PS_4_ groups can significantly affect the configurational energy landscape of this phase.

MC simulations show that Li starts to occupy Li2 (8e) sites at 200 K and Li3 (4c) sites at 300 K (Figure 6d). α-Li_3_PS_4_ thus begins to disorder at a much lower temperature compared to β-Li_3_PS_4_. By 600 K, Li atoms already occupy a significant fraction of each Li site, whereas β-Li_3_PS_4_ begins to disorder only at this temperature. Thus, the α polymorph contains much greater configurational disorder compared to β. The high configurational entropy of α leads to the experimentally observed isotropic Li fractional occupancies of around 0.4 for each site, as reported in ND refinements at 775 K (triangles in Figure 6d). We note that these ND-refined Li site occupancies deviate from the values obtained in our MC simulations (Figure 6d). This can potentially be attributed to the inability of our CE method to properly account for prominent PS_4_ rotational degrees of freedom in this phase, which may contribute to this CE having greater CV RMSE (4 meV/atom) compared to CE models of the other phases (Figure S6). More explicit treatment of strong lattice relaxations can in principle be done using adaptations of the CE method, such as the reciprocal space or rigid rotor CE formalisms.^41,42^

### Li_3_PS_4_ Phase Stability

**Figure 7:**
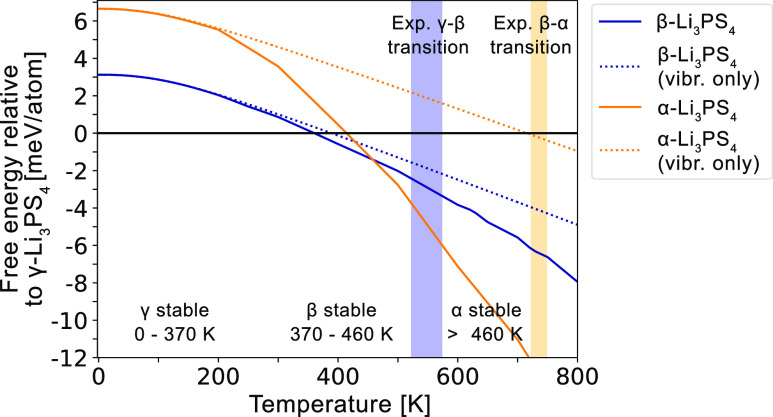
Free energy of α and β-Li_3_PS_4_ relative to γ-Li_3_PS_4_. All experimentally reported phase transitions are observed when accounting for all free energy contributions (solid lines). γ–β transition occurs at 370 K (523–573 K from exp), β–α at 460 K (723–748 K from exp), and α–γ at 420 K (533 K from exp upon cooling). Only the γ–β transition is observed when only vibrational entropy contributions are included (dotted lines). Shaded blue and orange regions indicate the experimentally observed γ–β and β–α phase transitions, respectively.

Using the structural models we validated for the Li_3_PS_4_ polymorphs, we assess the stability of each polymorph across temperature by calculating and comparing their free energy. In Figure 7, we plot the free energies of α and β relative to γ-Li_3_PS_4_. Since γ contains well-ordered Li, we assume it to only create vibrational entropy. At 0 K, the polymorphs ranked in order of decreasing stability are γ, β, and α. The γ–β transition is predicted to occur at 370 K, and the β–α transition occurs at 460 K (Figure 7). This order of phase transitions matches experiments, although the predicted transition temperatures are 200–300 K below experimentally observed values. In experiments, it is also commonly observed that α directly transforms to γ without forming β upon cooling,^11,14^ which we predict would occur at 420 K. At this temperature, the free energy differences among the polymorphs are very small (<1 meV/atom), which helps rationalize why a direct transition can occur, especially if the transformation to γ is more kinetically favorable than forming β. We note that the r^2^SCAN density functional^43^ is required to predict the correct order of Li_3_PS_4_ polymorph stability, since γ is predicted to be unstable across all temperatures when using PBE^44^ (Figure S1), which was also reported in previous first-principles calculations.^17^

When configurational entropy contributions are neglected (dotted lines in Figure 7), the free energy of α always lies above β, such that the only accessible transition is γ–β. This is attributed to the highly similar vibrational free energy profiles of α and β. After α begins to disorder at around 200 K, its configurational entropy increases faster than that of β, which drives the increased stability of α at high temperature. Furthermore, the exclusion of configurational entropy only slightly increases the γ–β transition temperature to 390 K, since β has a low configurational entropy at this temperature. The main source of stability for β-Li_3_PS_4_ is thus vibrational entropy.

**Figure 8:**
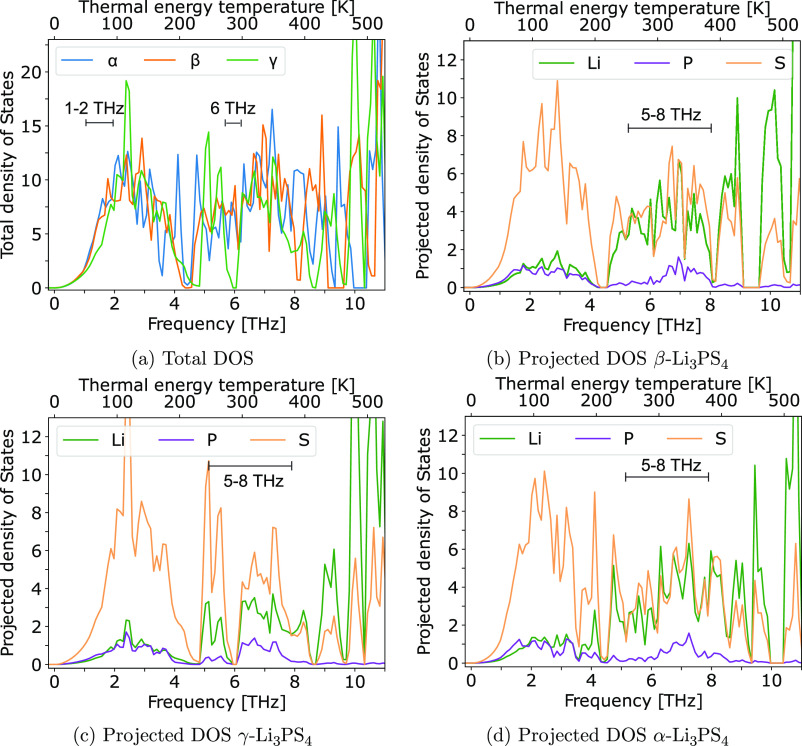
Phonon DOS. (a) TDOS for each Li_3_PS_4_ polymorph. PDOS onto Li, P, and S atoms for (b) β, (c) γ, and (d) α-Li_3_PS_4_ as a function of frequency and thermal energy temperature (*T* = *hf*/*k*_B_), normalized per unit cell of β-Li_3_PS_4_ (4 formula units). γ-Li_3_PS_4_ contains smaller DOS at frequencies of 1–2 and 6 THz. The region of 1–2 THz is dominated by S modes. At 5–8 THz, γ has a much larger density of S modes compared to Li, while in α and β there are equal contributions of each.

To rationalize the distinctly greater vibrational entropy in β and α compared to γ-Li_3_PS_4_, we compare the phonon density of states (DOS) in each phase, which are shown in Figure 8a. β and α-Li_3_PS_4_ contain significantly larger total DOS (TDOS) at 1–2 THz and around 6 THz (Figure 8a). The projected DOS (pDOS) shows that for all phases, the 1–2 THz region is dominated by sulfur (S) modes, which are activated at low temperature around 100 K. From visualizing these modes, we observe that they mainly correspond to librations of the PS_4_ groups. Furthermore, γ has no vibrations at 6 THz, whereas the high-temperature phases contain significant DOS near this frequency. This frequency lies in the region between 5 and 8 THz (240 to 380 K), where β (Figure 8b) and α (Figure 8d) have roughly equal projected density of Li and S phonon modes, whereas in γ there is a significantly larger projected density of S modes than Li (Figure 8c). The activation of larger amplitude Li modes at around room temperature contributes to greater thermodynamic stability and potentially toward high Li mobility in β and α-Li_3_PS_4_. This finding is consistent with prior reports highlighting the relation between fast Li-ion conductivity and vibrational entropy in some superionic conductors.^45^

**Figure 9:**
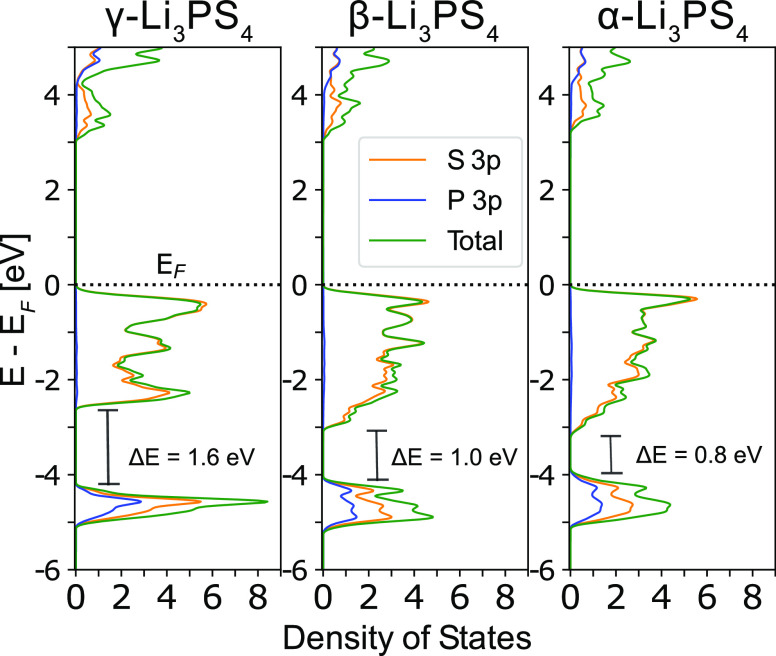
Orbital-resolved eDOS of the γ, β, and α-Li_3_PS_4_ ground-state structures. Energies are referenced to the Fermi level (*E*_F_). All phases exhibit a valence band manifold consisting of mainly S 3p states and a lower energy core band manifold composed of hybridized S 3p and P 3p states. γ-Li_3_PS_4_ has a larger energy gap (Δ*E*) between valence and core band manifolds (1.6 eV) compared to those of α and β (0.8 and 1.0 eV), as well as a sharper eDOS peak in the core manifold.

Since the differences in low-temperature vibrational modes are likely dictated by the bonding within the S sublattice, we examine the electronic DOS (eDOS) of each ground state, which are shown in Figure 9. For all three polymorphs, the manifold of valence bands below the Fermi level dominantly consists of S 3p states which are spread over an energy range of ∼3 eV. The relatively large band widths indicate that these states are delocalized in character and should represent long-range van der Waals interactions among S atoms in separate PS_4_ units (Figure 9). The lower energy core band manifold is mainly composed of P 3p and S 3p states, which we attribute to the P–S hybridization between the PS_4_ groups. These core band states are spread over a narrower energy range of ∼1 eV, indicating that these states are more localized.

Key differences in electronic structure are observed in the γ polymorph, which has a larger energy gap between the core and valence band states, arising from narrower band widths in the core and valence band manifolds (Figure 9). The core band manifold of γ also exhibits a distinctly sharp peak in the eDOS at around −4.5 eV, which results from flatter core bands. The narrower core band widths can arise from stronger hybridization of S 3p and P 3p states in neighboring PS_4_ units, leading to more localization. This stronger hybridization between S and P atoms may lead to smaller interaction between S 3p states on neighboring PS_4_ groups, which contributes to decreased valence band widths. It appears that the unidirectional PS_4_ arrangement and denser hcp-type anion packing in γ-Li_3_PS_4_^14^ facilitate more isotropic and localized P–S bonding states to inhibit facile S motion. These factors would contribute to greater S sublattice stiffness and reduced density of low-frequency S vibrational modes.

### Li_7_PS_6_ Polymorphs

**Figure 10:**
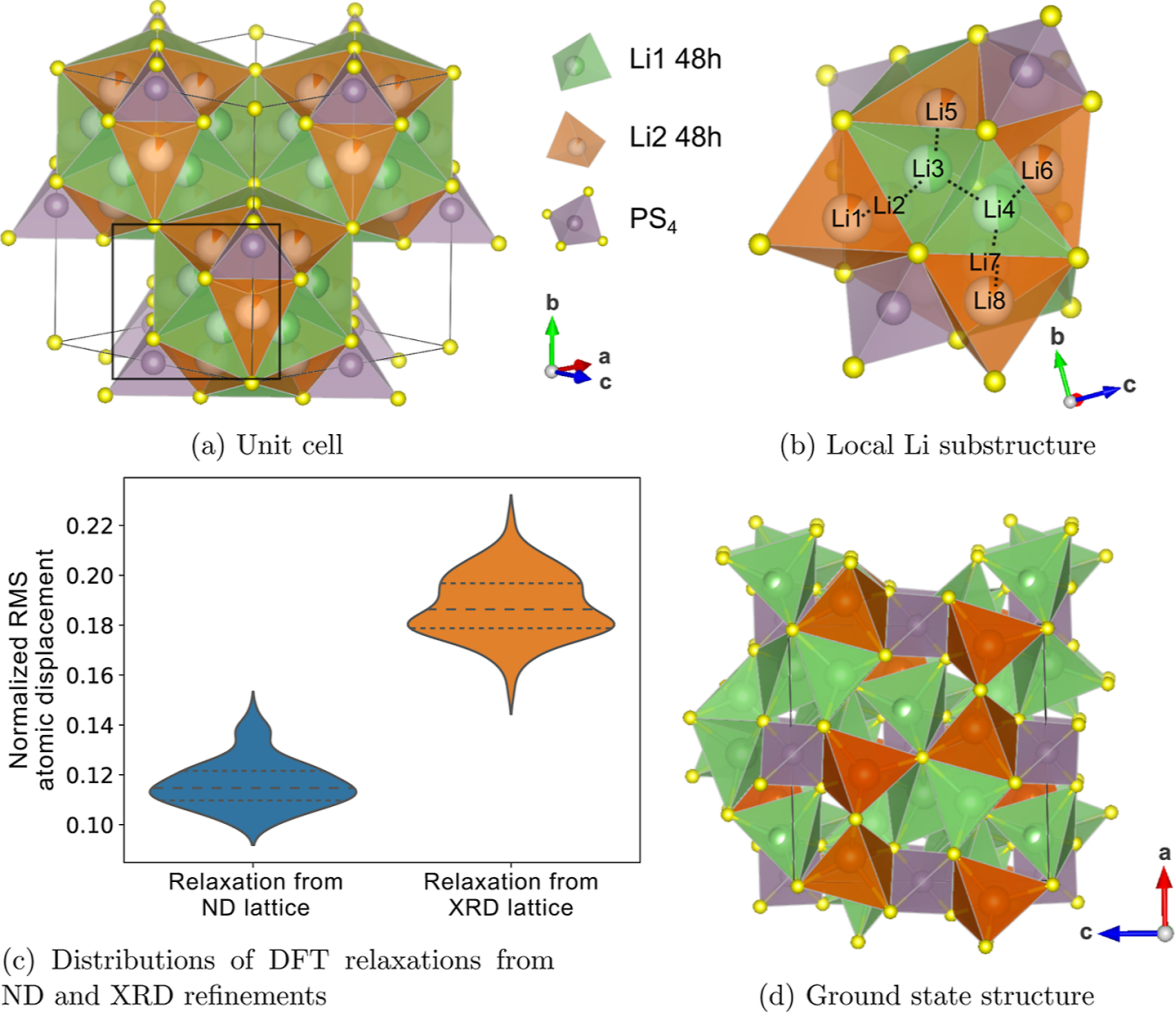
Structure of HT-Li_7_PS_6_. (a) The unit cell (*F*4̅3*m*) with Li1 (48h) (green) sites that are corner-sharing with PS_4_ and Li2 (48h) (orange) sites that are edge-sharing with PS_4_. The boxed region containing a single cage-like Li substructure is shown in greater detail in (b), which displays the local Li site coordination. Dotted lines connect the face-sharing Li atoms. (c) Violin plots displaying distributions of NRMS atomic displacements of DFT-relaxed configurations from ND and XRD refinements. Dashed lines denote a division between data quartiles. (d) Ground-state structure of HT-Li_7_PS_6_, containing 6 occupied Li2 (48h) sites (orange).

Experiments show that orthorhombic LT-Li_7_PS_6_ (*Pna*2_1_) is well-ordered and transforms to the higher symmetry cubic HT-Li_7_PS_6_ (*F*4̅3*m*) phase at 483 K.^12^ According to XRD refinements, HT-Li_7_PS_6_ contains a disordered Li sublattice with one distinct Li1 (48h) site, which is corner-sharing with PS_4_ units and face-sharing with its nearest Li1 neighbor.^12^ No ND refinement has yet been reported on the HT-Li_7_PS_6_ phase; however, ND refinements have been reported for a Cl-doped analogue Li_6_PS_5_Cl.^19^ An additional Li2 (48h) site was identified in Li_6_PS_5_Cl that is edge-sharing with PS_4_ units and face-sharing with its nearest Li1 and Li2 neighbors to form a cage-like Li substructure (Figure 10b), while the sublattices of the PS_4_ and isolated S atoms are identical. Since Cl substitutes a fraction of S atoms without causing much change in lattice parameters, we presume that the Li sites in the doped and pristine phases are very similar and comparable.^19^

As done with the β-Li_3_PS_4_ phase, we compare the NRMS atomic relaxations (eq 2) of the DFT-relaxed configurations starting from either the ND- or XRD-refined atomic positions of HT-Li_7_PS_6_, the distributions of which are plotted in Figure 10c. We observe that there is a much smaller NRMS atomic displacement from the ND-refined lattice (a mean of 0.12) compared to the XRD-refined lattice (mean of 0.19), indicating that the ND positions for Li are closer to the energy minimum.

We model Li-vacancy disorder in HT-Li_7_PS_6_ by fitting a CE using the ND refinement of Li_6_PS_5_Cl containing Li1 (48h) and Li2 (48h) sites, with all Cl atoms replaced by S atoms. Through MC simulated annealing, we identify a ground-state ordering, shown in Figure 10d, which contains 6 Li atoms in the unit cell (out of 28 Li) occupying Li2 sites, as evidenced by their edge-sharing with PS_4_ (orange in Figure 10d). The prominence of Li2 as a stable site in the ground state provides further evidence that the structure refined by ND is more accurate and that Cl doping does not influence the location of Li sites.

**Figure 11:**
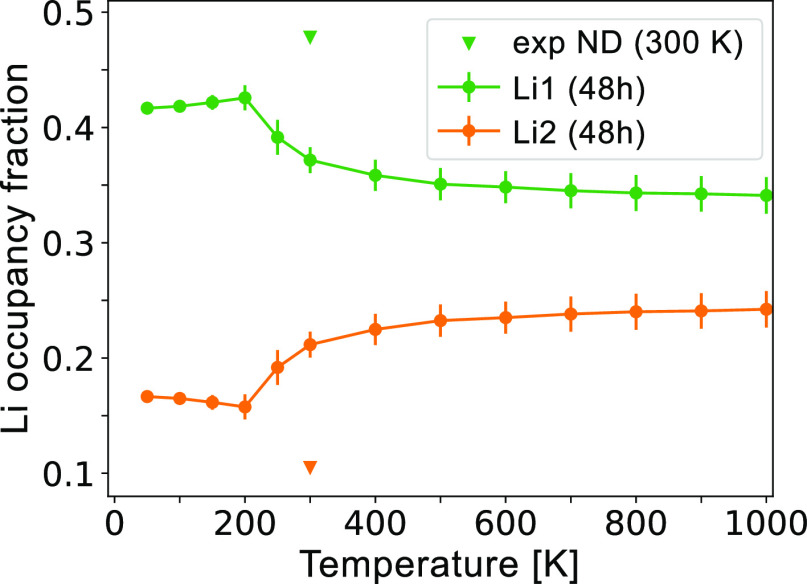
HT-Li_7_PS_6_ Li site occupancy fractions across temperature. The reported experimental occupancies (triangles) were measured with ND on Li_6_PS_5_Cl at 300 K, and they are scaled by 7/6 to account for the different Li stoichiometry, compared to Li_7_PS_6_.

We perform MC simulations to predict the Li site occupancies as a function of temperature, which are plotted in Figure 11. The fraction of Li occupying Li1 is greater at all simulated temperatures, in reasonable agreement with Li site occupancy of Li_6_PS_5_Cl measured by ND at ambient temperature.^19^ The preference of Li going to Li1 sites could be explained by its corner-sharing with PS_4_, which can reduce the repulsive interaction with P cations compared to the edge-sharing Li2 sites.^46^

**Figure 12:**
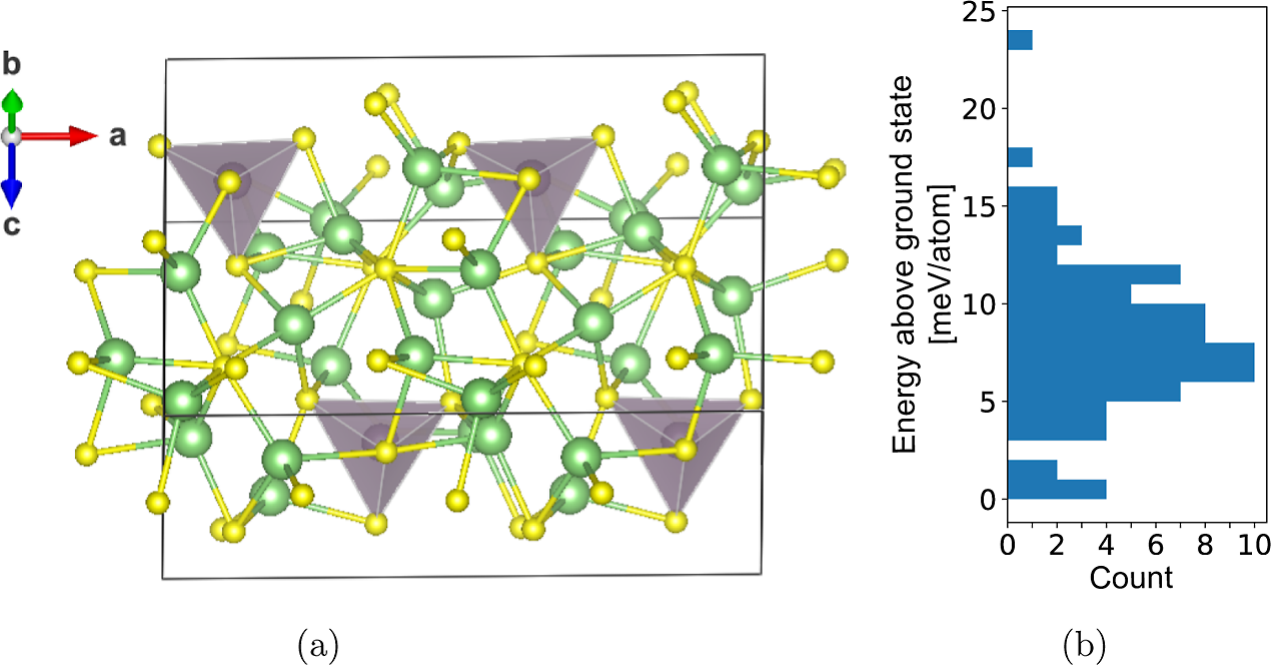
Structure of LT-Li_7_PS_6_. (a) Ground-state structure identified from AIMD simulations. PS_4_ units relax slightly away from a parallel arrangement, contributing to a slight monoclinic distortion, with unit cell angles: (α, β, γ) = (90.0, 91.0, 90.0). (b) Histogram of energies above ground state for structures sampled along the AIMD trajectory. Reported energies are calculated from ionic relaxations using the PBE functional.

The HT-Li_7_PS_6_ ground-state structure was found to be 10.4 meV/atom more stable than the ordered LT-Li_7_PS_6_ structure proposed by XRD (shown in Figure S2), suggesting that the XRD refinement for LT-Li_7_PS_6_ may not be accurate.^12^ To seek a more representative LT-Li_7_PS_6_ structure, we perturb its XRD-refined structure through an AIMD simulation. The structure is heated to 800 K for 2 ps, held for 30 ps, and annealed to 100 K for 20 ps. Samples along the AIMD trajectory are relaxed, from which we identify a significantly more stable structure that is 1.2 meV/atom below the HT-Li_7_PS_6_ ground state. This new LT-Li_7_PS_6_ ground state (shown in Figure 12a) has a slight monoclinic distortion (lattice angle β = 91°), resulting from a small relaxation of the PS_4_ units away from a parallel arrangement, and some Li are shifted to new coordination environments. We also find a large spread of energies among the sampled structures that were relaxed (Figure 12b), indicating that LT-Li_7_PS_6_ is likely configurationally disordered as well. We leave further analysis of the LT-Li_7_PS_6_ Li sublattice for future investigation. However, our investigation confirms that, with our reassignment of the Li sites, LT-Li_7_PS_6_ is the ground state at low temperature.

**Figure 13:**
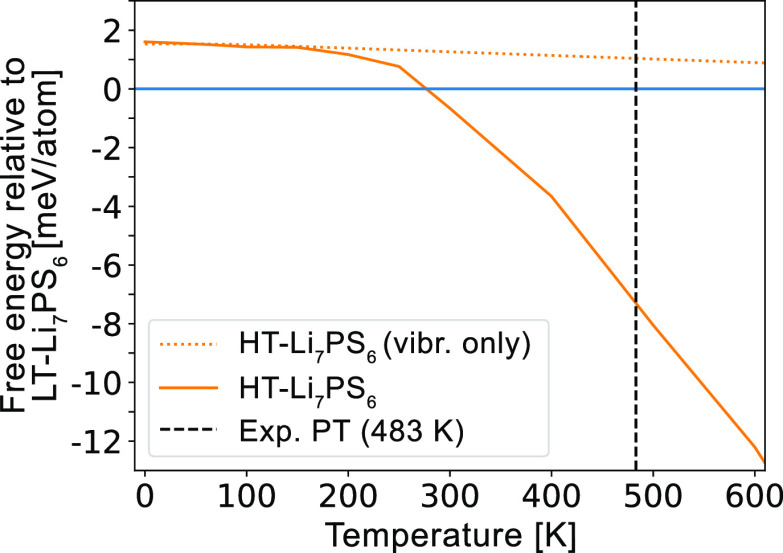
Free energy of HT-Li_7_PS_6_ relative to LT-Li_7_PS_6_. Phase transition from LT to HT is predicted at 280 K, compared to 483 K from experiment.

Using our newly proposed ground states, we predict the phase stability of the Li_7_PS_6_ polymorphs at finite temperatures. In Figure 13, we plot the free energy of HT-Li_7_PS_6_ relative to LT. HT-Li_7_PS_6_ becomes stable at 270 K, with the majority of its stabilization relative to that of LT-Li_7_PS_6_ arising from configurational entropy contributions (Figure 13). Our predicted transition temperature is roughly 200 K below its experimentally observed value of 480 K. The likely cause for this understabilization of LT-Li_7_PS_6_ is that we may not have identified its true ground state yet and that it contains significant configurational entropy contributions that have been neglected from our model because of the lack of a precise Li sublattice.

### Li_7_P_3_S_11_

Li_7_P_3_S_11_ crystallizes in the low symmetry *P*-1 space group and is composed of PS_4_ and P_2_S_7_ units. In both XRD and ND refinements, the Li sublattice is ordered with 7 distinct sites.^13,31^ However, each refinement reports Li atoms occupying a different set of sites (the structures are shown in Figure S4). From our DFT calculations, we find that the XRD-refined structure is substantially more stable than the ND-refined structure by 9 meV/atom. A more recent first-principles study by Chang and co-workers proposed a disordered Li sublattice with 8 additional Li sites, identified from AIMD simulations.^32^ The authors enumerated structures based on the disordered Li sublattice and reported a ground state (Figure S4c) that is 0.8 meV/atom more stable than the XRD-refined structure using the PBE functional. This value is qualitatively consistent with our calculations using r^2^SCAN, which yield an energy difference of 1.0 meV/atom.

**Figure 14:**
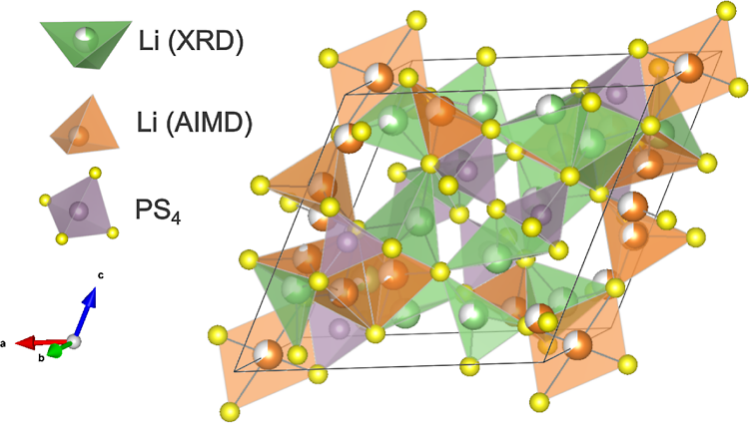
Disordered unit cell of Li_7_P_3_S_11_ with 7 distinct Li sites identified from XRD (green) and 8 distinct sites identified from AIMD simulations (orange).

We train a CE on the previously reported disordered Li_7_P_3_S_11_ lattice containing 15 distinct Li sites, which are a sum of the sites identified from XRD and AIMD. The unit cell is shown in Figure 14, from which we can observe that the possible Li sites include a range of planar and tetrahedral coordination environments with varying degrees of distortion. Through MC simulated annealing, we uncover a new ground-state ordering (Figure S4d) that is 1.0 meV/atom more stable than the ground state previously proposed by Chang and co-workers.^32^

**Figure 15:**
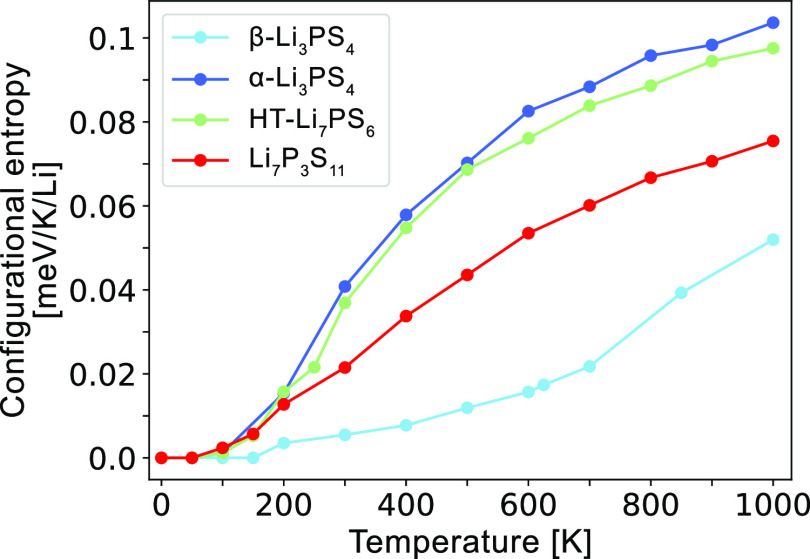
Configurational entropy in the disordered superionic conductors α-Li_3_PS_4_, β-Li_3_PS_4_, HT-Li_7_PS_6_, and Li_7_P_3_S_11_ phases, normalized per Li atom.

Since crystallographic refinements have not reported the existence of configurational disorder in this phase, it is important to quantify the degree of disorder and compare this with other superionic conductors.^13,31^ To that end, we calculate the configurational entropy as a function of temperature with MC simulations for Li_7_P_3_S_11_ and compare it to that of α-Li_3_PS_4_, β-Li_3_PS_4_, and HT-Li_7_PS_6_, which are plotted in Figure 15. Li_7_P_3_S_11_ (red) is predicted to contain significant configurational entropy that is greater than β-Li_3_PS_4_ (light blue) and lower but comparable to α-Li_3_PS_4_ (dark blue) and HT-Li_7_PS_6_ (green). This result corroborates the additional Li sites in the disordered Li sublattice identified from AIMD.^32^ We remark that all superionic conductors in this phase space contain significant configurational entropy that is of the same order of magnitude, indicating a potential correlation between superionic conductivity and configurational entropy.

## Discussion

Through first-principles calculations, we derive a phase diagram of the Li_2_S–P_2_S_5_ system that recovers well-established experimental trends such as polymorph phase transitions in Li_3_PS_4_ and Li_7_PS_6_, and the instability of Li_7_P_3_S_11_ at high temperature. All superionic conductors are predicted to be metastable at room temperature. An accurate assessment of the configurational entropy required precise information about the possible Li sites in these structures. We find that ND refinements tend to contain more accurate details about Li sites and degree of disorder compared to XRD refinements. Our calculations show that these details from ND are critical toward predicting physically accurate dynamical stability and thermodynamic behavior.

We predict that all superionic conductors in this space are metastable at ambient temperature, in agreement with previous studies.^5,9,10,14,20,23^ Specifically, α-Li_3_PS_4_, β-Li_3_PS_4_, HT-Li_7_PS_6_, and Li_7_P_3_S_11_ have energies above the hull (*E*_hull_) of 4, 1, 12, and 4 meV/atom at 300 K, respectively. These values are well within the range of energy for which metastability is observed in the large-scale study on metastability by Sun et al., who found that metastable inorganic sulfide compounds in the inorganic crystal structure database yielded a median *E*_hull_ of 9.7 meV/atom and a 90th percentile *E*_hull_ of 45.3 meV/atom at 0 K.^35^ HT-Li_7_PS_6_ is the only superionic conductor in this system with greater *E*_hull_ at 0 K (16 meV/atom) than the median value, and all are below the 90th percentile value found by Sun et al.^35^ While low *E*_hull_ has been suggested to be a required criterion for metastability, Sun et al.^35^ point out that it is not sufficient to guarantee metastability. However, the low *E*_hull_ values for these phases are certainly consistent with their accessibility.

Vibrational and configurational sources of entropy are shown to be crucial toward describing phase stability trends. Among the Li_3_PS_4_ polymorphs, the superionic conductors α and β have distinctly greater vibrational entropy compared to γ, which has low Li conductivity. We attribute this to the softness of the anion sublattice, as α and β-Li_3_PS_4_ contain significantly more low-frequency S vibrational modes, mainly corresponding to librations of the PS_4_ group. The potential electronic origin of the stiffer anion sublattice in γ-Li_3_PS_4_ lies in the stronger hybridization of the P 3p and S 3p states near the Fermi level. We postulate that these subtle differences in longer range binding between PS_4_ units are the reason why a meta-GGA level of theory is required to predict the correct order of Li_3_PS_4_ polymorph stability, as the SCAN family of density functionals have been shown to be superior at capturing medium-range van der Waals interactions.^47,48^ These findings can potentially motivate new design principles for novel superionic conductors based on features of the phonon and electronic band structure.^45^

Configurational sources of entropy are also essential toward describing phase stability trends. The polymorphic phase transitions involving α-Li_3_PS_4_ and HT-Li_7_PS_6_ can only be predicted when accounting for configurational disorder, which in turn requires accurate assessment of possible sites that Li can access. Furthermore, all superionic conductors in this phase space generate a significant amount of configurational entropy. β-Li_3_PS_4_ has the lowest configurational entropy, and coincidentally its bulk ionic conductivity has been reported to be low (8.9 × 10^–3^ mS/cm), with only its nanoporous form having high Li conductivity (0.16 mS/cm).^15^ The high-temperature α polymorph has considerably greater configurational entropy and a room-temperature Li conductivity of ∼2 mS/cm.^18^ Meanwhile, the γ polymorph has the lowest ionic conductivity and contains no configurational disorder. This observation suggests an inherent correlation between fast Li mobility and high configurational entropy.

This trend is observed in many other systems as well. We show that HT-Li_7_PS_6_ has high configurational entropy, comparable to α-Li_3_PS_4_, and it is experimentally shown to have greater Li conductivity than LT-Li_7_PS_6_.^20^ This trend is not unique to sulfide superionic conductors, as the oxide garnet Li_7_La_3_Zr_2_O_12_ (LLZO) has a low-temperature ordered tetragonal phase with low Li conductivity, and a high-temperature disordered superionic conductor with increased cubic symmetry.^49^ We observe that superionic conductors tend to be high-temperature polymorphs with increased symmetry arising from the configurational disorder. These phases must be entropically stabilized at high temperature, which lends further support that high entropy is favorable toward achieving a superionic conducting state.

**Figure 16:**
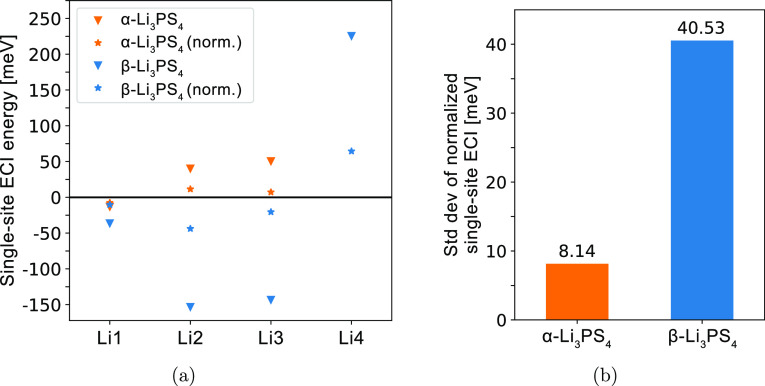
Comparing Li site energies in α- and β-Li_3_PS_4_ polymorphs. (a) ECI energies corresponding to single-site functions in the CE (*J*_ECI_, denoted by triangles), and ECI energies normalized by site multiplicity (, denoted by stars). In β, the sites are Li1 (8d), Li2 (8d), Li3 (4c), and Li4 (8d). Positive values correspond to an increase in energy from Li occupying a site. In α, the sites are Li1 (16h), Li2 (8e), and Li3 (4c). (b) Standard deviation of .

We can rationalize the origin of the high configurational entropy by analyzing Li site energies. β-Li_3_PS_4_ and its higher symmetry α-Li_3_PS_4_ polymorph are ideal systems to compare, as they have the same number of Li atoms and Li sites per unit cell. A first-order approximation for the Li site energy is the site’s ECI energy (*J*_ECI_) obtained from the CE using an orthonormal basis, which are plotted in Figure 16a. It can be shown from the cluster decomposition framework that this is a unique and physical value to describe the energy of Li occupying a particular site.^50^ This approximation can be justified by the observation that single-site ECI tend to be much larger in magnitude than the multisite pair and triplet ECI (Figure S5); single-site ECI thus carry most of the weight in the total energy. We also calculate a site energy normalized by its multiplicity (), which would provide a better estimate of the energy contribution of the site per unit cell. This is described in eq 3, where *M* is the multiplicity of a distinct Li site and *N* is the total number of Li sites per unit cell.
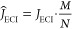
3In Figure 16b, we plot the standard deviation of  in each Li_3_PS_4_ phase, showing that α contains a significantly smaller spread of  (8 meV) compared to the β polymorph (41 meV). Thus, in α, the Li atoms will have a comparable energetic preference for occupying all sites. Many configurations will then have similar energy, which contributes toward its greater configurational entropy. The larger Li site energy spread in β means that Li atoms will tend to order by occupying the lowest energy sites and thus have smaller configurational entropy.

The potential connection between high Li mobility and configurational entropy suggests that increasing disorder should be a promising strategy to design superionic conductors. Indeed, there have been many examples where aliovalent cation or anion doping improves the Li conductivity and enables room-temperature phase stability. These include adding Si into Li_3_PS_4_ to form Li_3.25_P_0.75_Si_0.25_S_4_ in the β-Li_3_PS_4_ structure,^17^ adding Cl or other halogen atoms (X) to Li_7_PS_6_ to form Li_6_PS_5_X in the HT-Li_7_PS_6_ structure,^19,21,22^ and doping Al or Ga into Li_7_La_3_Zr_2_O_12_ to stabilize its high-temperature cubic structure.^51^ In some cases, it has been claimed that inducing alkali-ion vacancies even without the presence of dopants can stabilize superionic conducting polymorphs, such as the high-temperature Na_3_PS_4_ polymorph.^52^ Our MC simulations have shown that the disorder arising from only Li and vacancies can generate substantial configurational entropy and that this entropy can dictate phase stability trends.

Previous studies have also shown that adding dopant species can alter the Li site energy landscape to facilitate dramatic improvements in the ionic conductivity. Zeng and co-workers demonstrated that high-principal element cation doping can boost ionic conductivity by orders of magnitude.^53^ Through first-principles calculations, they showed that distortions to Li environments introduced by dopants can lead to Li site energy levels that are more closely spaced, promoting Li-ion percolation. It is possible that the soft degrees of freedom for libration of the PS_4_ units as seen in several polymorphs further generate the distribution of temporary site energies which leads to low energy barrier percolation pathways.^54^ Similarly, Wang and co-workers found that adding Br into Li_3_YCl_6_ to form Li_3_YBr_1.5_Cl_4.5_ introduced a larger variety of closely spaced octahedral Li site energy levels, leading to a lower order–disorder transition temperature and increased Li conductivity.^55^ These previous studies highlight that engineering a more uniform Li site energy landscape can facilitate more facile Li-ion migration since there is a greater probability that neighboring sites are close in energy, which in turn can lead to lower energy barriers for Li hopping between them. This is not a sufficient condition for high conductivity, as the component of the migration energy that is independent of the site energy also needs to be small. We can synthesize this with our finding that a smaller variance in Li site energies necessarily leads to greater configurational disorder as well, which illustrates why the phenomena of superionic conductivity and high configurational entropy should be intrinsically linked. This rationalizes why tuning the Li site energy landscape through introducing dopants and Li off-stoichiometry has been and should continue to be an essential design principle for discovering superionic conductors with improved Li conductivity and thermodynamic accessibility.

Accurately modeling configurational disorder in each phase could only be achieved after clarifying the details of the Li sublattices. We demonstrate that ND refinements of Li sublattices in α-Li_3_PS_4_, β-Li_3_PS_4_, and the Li_6_PS_5_Cl analogue of HT-Li_7_PS_6_ contain critical details such as site splitting and additional sites that XRD could not detect. These additional sites likely lead to more low-energy configurations that are vital for describing thermodynamic behavior. The deficiencies of XRD refinements can be attributed to Li having poor XRD sensitivity due to its small X-ray scattering factor, while the negative scattering length of Li neutrons leads to greater sensitivity in ND. Despite its known limitations, XRD often yields reasonable results in many Li-containing materials, such as Li transition metal oxide cathodes, and remains a standard technique in characterizing Li battery materials. We speculate that the spurious XRD refinements highlighted in this study stem from very high Li mobility, which would smear the detected Li electron density and thus further deteriorate sensitivity. The close agreement between ND and XRD refinements of γ-Li_3_PS_4_ can then be explained by its low Li conductivity.^11^ Our discovery of configurational disorder in LT-Li_7_PS_6_ highlights that there may still be additional details about the Li substructures that have yet to be uncovered, which should motivate further experimental and computational studies to refine the Li atomic arrangements.

Although we have predicted the phase stability trends and rationalized them on the basis of configurational and vibrational contributions, our predicted phase transition temperatures tend to underestimate experimentally observed values by about 200 K. The phase stability trends in this system are described on a rather fine energy scale on the order of 10 meV/atom. Subtle changes such as hypothetically shifting the free energy curve of β-Li_3_PS_4_ up by 3 meV/atom can already increase the γ–β transition temperature to its experimentally observed window. These small energy differences are easily within the bounds of error in our computational techniques.

Specifically, it is known that semilocal density functionals, such as the PBE and r^2^SCAN functionals, struggle to capture long-range dipole–induced dipole interactions,^47^ which are likely to be prominent within the S sublattice in these materials. Furthermore, there is remnant self-interaction error in density functional approximations,^56^ which can be mitigated by using more computationally expensive hybrid functional techniques^57^ or many-body treatments of electron correlation.^58^ The error from CE configurational energies is compounded onto the DFT error, since the CEs are trained on DFT data. On the basis of CV RMSE, CE energy error ranges from 1 to 5 meV/atom, depending on the phase (Figure S6). Furthermore, anharmonic corrections to phonon calculations may yield key differences in the band dispersion and resulting vibrational free energy, as previously demonstrated in the sodium thiophosphate (Na_3_PS_4_) analogue.^59^ The facile and long-range nature of Li hopping modes is a potential source of anharmonicity in superionic conductors. Finally, we have treated the configurational and vibrational entropy contributions as independent, as is common in first-principles alloy theory.^60^ A more accurate, but significantly more computationally intensive approach, would also be to include the configurational dependence of the vibrational entropy, as can be formally done with the CE approach.^61,62^

## Conclusions

A phase diagram of the pseudo-binary Li_2_S–P_2_S_5_ system has been constructed from first-principles calculations. Well-established experimental trends, such as the phase transitions among Li_3_PS_4_ and Li_7_PS_6_ polymorphs, and the metastability of Li_7_P_3_S_11_ are recovered. The superionic conductors α-Li_3_PS_4_, β-Li_3_PS_4_, HT-Li_7_PS_6_, and Li_7_P_3_S_11_ are all predicted to be metastable at 300 K (*E*_hull_ = 4, 1, 12, and 4 meV/atom, respectively) but thermodynamically accessible. We find that accounting for both vibrational modes and Li configurational disorder is essential for describing phase stability trends. Physically accurate evaluation of configurational entropy could only be made after clarifying the details of the Li sublattices in the superionic conductors. We demonstrate that these phases all contain significant configurational entropy, which suggests a correlation between high Li configurational entropy and fast Li conduction. Engineering a more uniform Li site energy landscape through doping and tuning the Li content should thus be essential design principles for discovering novel superionic conductors with improved thermodynamic stability and Li conductivity at ambient temperature.

## Methods

All electronic structure calculations were performed using the Vienna ab initio simulation package (VASP).^63^ For the ground-state structures of each phase, ionic relaxations were performed with 1 × 10^–5^ eV convergence in the total energy and 1 × 10^–2^ eV/Å in the forces, initially using the generalized gradient approximation (GGA) functional as parametrized by Perdew, Burke, and Ernzerhof (PBE),^44^ projector augmented wave pseudopotentials,^64^ and a plane-wave basis set with an energy cutoff of 520 eV. The GGA-converged structure was further relaxed with the meta-GGA r^2^SCAN functional,^43^ with a *k*-point spacing dependent on the band gap of the PBE calculation, a scheme proposed by Kingsbury and co-workers.^65^ The final reported formation energies were obtained from a static calculation with a denser *k*-point spacing of 0.2 Å^–1^. To plot the eDOS, we perform another static calculation using r^2^SCAN with stricter electronic convergence (1 × 10^–7^ eV) and the eDOS was calculated at 2000 energy points. Applying increased meta-GGA level of theory was essential for capturing physical polymorph phase stability, as γ-Li_3_PS_4_ and β-Li_3_PS_4_ had nearly identical electronic formation energies using PBE (Table SI). The relaxed structure, total energy, and calculation details for each phase’s ground state are attached in the form of a Pymatgen ComputedStructureEntry JSON file in the attached lpṡfinal̇gṡentries.zip folder.^37^ All structure generation, manipulation, and symmetry analyses were performed using Pymatgen.^37^

Cluster expansion (CE) construction and Monte Carlo (MC) sampling were performed with the smol Python package.^66^ The primitive structures used to construct the CE for each phase are described in Tables SII–SV. CEs were trained on superstructures relaxed using the PBE functional only to limit the computational cost. It has been previously shown that similar schemes of mixing levels of theory can yield physically accurate phase diagrams.^67^ We simultaneously parametrize the CE with an additional electrostatic term to describe long-range Coulomb interactions. To that end, we fit an effective dielectric constant (ϵ) that screens the ionic electrostatic energy, which was calculated from the bare Coulomb interaction between idealized Li^+^, P^5+^, and S^2–^ point charges with the Ewald summation method as implemented in Pymatgen.^37^ CE fitting was performed in a piece-wise manner, where the initial fit only trained the point correlation functions and effective dielectric constant (ϵ), using L_2_ norm penalized linear regression.^68^ The residual of the initial fit was used to train the pairs and higher-order ECI with penalization of the L_1_ norm to promote a sparse solution.^69,70^ We observed that this method yields improved fit stability and a more physical ϵ, which is attributed to decreased regularization of ϵ.^25^ The optimal regularization hyperparameters were chosen to minimize the cross-validation (CV) root-mean-squared error (RMSE), with the condition that the training set RMSE lies within the standard deviation of the CV RMSE, to mitigate overfitting. MC sampling was performed in supercells for each phase in the canonical ensemble with decreasing temperatures starting at 1000 K. Supercells were constructed to contain at least 200 Li sites and have similar lattice parameters. At least 40,000 MC passes were performed at each temperature. To calculate configurational free energy, the average internal energy (⟨*E*⟩) at each temperature was integrated over inverse thermal energy () (eq 4).
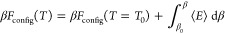
4New ground states were found from simulated annealing, using a similar procedure of canonical MC sampling at decreasing temperature but with unit cells and smaller supercells.

Harmonic phonon calculations were performed on the ground state of each phase, with the frozen phonon method using Phonopy and VASP.^34^ Structures were relaxed with PBE to stricter convergence criteria of 1 × 10^–7^ eV in energy and 1 × 10^–3^ eV/Å in the forces. Atomic displacements were generated on supercells, which were created such that each lattice parameter is greater than 12 Å and nearly equal to each other. For P_2_S_5_ only, we calculate the phonon properties using density functional perturbation theory (DFPT) as implemented in VASP^71^ because we observed that the frozen phonon method yielded many imaginary modes, which we attribute to strong anharmonicity. Nonanalytical correction to modes near the Γ wave vector was performed by incorporating the dielectric properties, to account for longitudinal optical and transverse optical (LO-TO) mode splitting in polar ionic materials in the long wavelength limit.^72^ Dielectric permittivity and Born effective charge tensors were computed with DFPT,^71^ using a denser reciprocal space discretization of 0.125 Å^–1^ to ensure convergence of dielectric properties.^73^ The vibrational free energies and phonon total density of states (TDOSs) for each phase (if not already shown in the previous sections) are plotted in Figures S8 and S9, respectively.

Using the electronic, configurational, and vibrational free energies, the formation free energies and resulting phase diagrams were computed with Pymatgen.^37^ The formation free energies of phases relative to the Li_2_S and P_2_S_5_ end points are shown in Figure S7.

## Supporting Information Available

The relaxed structure, total energy, and calculation details for each phase’s ground state structure (ZIP)
Comparing phase stability results of Li_3_PS_4_ polymorphs obtained from PBE and r^2^SCAN functionals, structures of LT-Li_7_PS_6_ and Li_7_P_3_S_11_, details of CE construction, CE training errors, formation free energies, and vibrational properties of each phase’s ground-state structure (PDF)
