# Supplementary material for: Crystal Structures and Phase Stability of the Li2S–P2S5 System from First Principles
Source: Chem Mater. 2023 Oct 23;35(21):9111–26. doi: 10.1021/acs.chemmater.3c01793 (PMC10653090; doi:10.1021/acs.chemmater.3c01793)
Supplement: Supplementary file 2 — cm3c01793_si_002.pdf [file cm3c01793_si_002.pdf]

Supplementary Information: Crystal structures and phase stability of the Li_2S-P_2S_5 system from first principles

Ronald L. Kam,[†,‡] KyuJung Jun,[†,‡] Luis Barroso-Luque,[†] Julia H. Yang,[†,¶] Fengyu Xie,[†,‡] and Gerbrand Ceder[*,†,‡]

†*Materials Science Division, Lawrence Berkeley National Laboratory*

‡*Department of Materials Science and Engineering, University of California, Berkeley*

¶*John A. Paulson School of Engineering and Applied Science, Harvard University (current affiliation)*

E-mail: gceder@berkeley.edu

I: Phase stability of Li_3PS_4 polymorphs

TABLE SI: Phase stability of Li_3PS_4 polymorphs at 0 K. Energies relative to γ-Li_3PS_4 [meV/atom] are calculated using PBE and r^2SCAN functionals, with and without the zero point vibrational energy. Zero point vibrational energy is calculated using the frozen phonon method with PBE.

Polymorph	PBE	PBE+zero pt	r^2SCAN	r^2SCAN+zero pt
β	-0.3	-2.3	5.1	3.1
α	1.6	-0.1	8.4	6.6

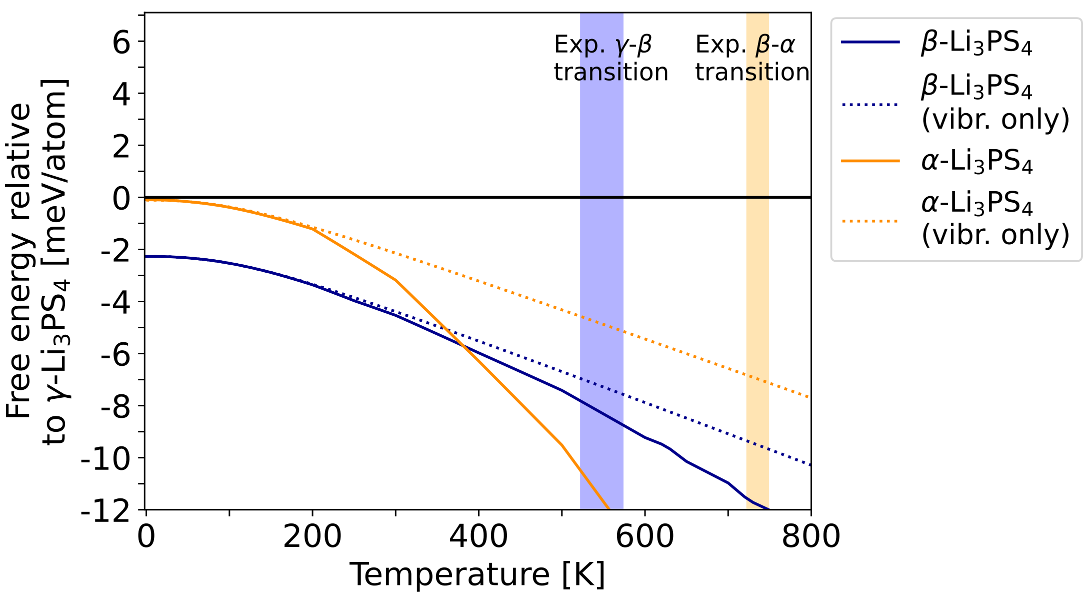

Figure S1: Free energy of α amd β-Li_3PS_4 relative to γ-Li_3PS_4, calculated with the PBE functional. The γ polymorph is predicted to be unstable across all temperatures. Shaded blue and orange regions indicate experimentally observed γ-β and β-α phase transitions, respectively.

II: LT-Li_7PS_6

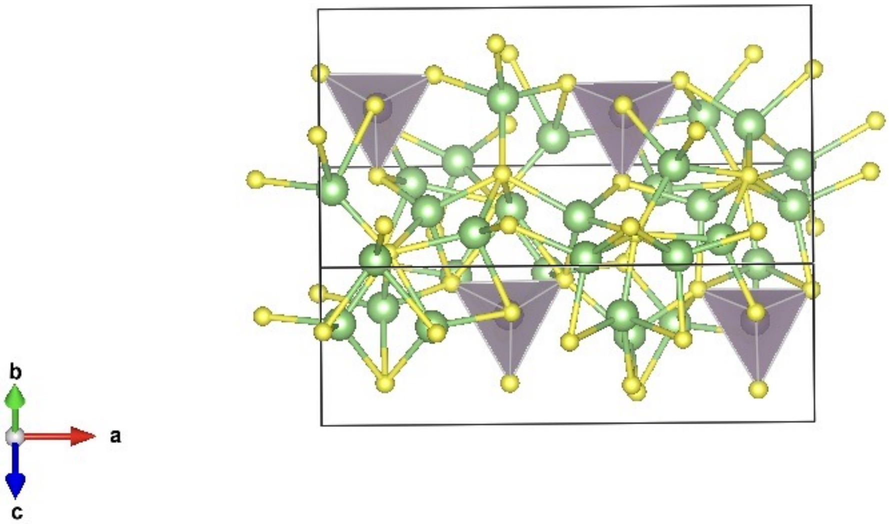

Figure S2: XRD refinement of LT-Li_7PS_6 ($Pna2_1$),[1] relaxed with r^2SCAN.

III: $Li_7P_3S_{11}$ low energy structures

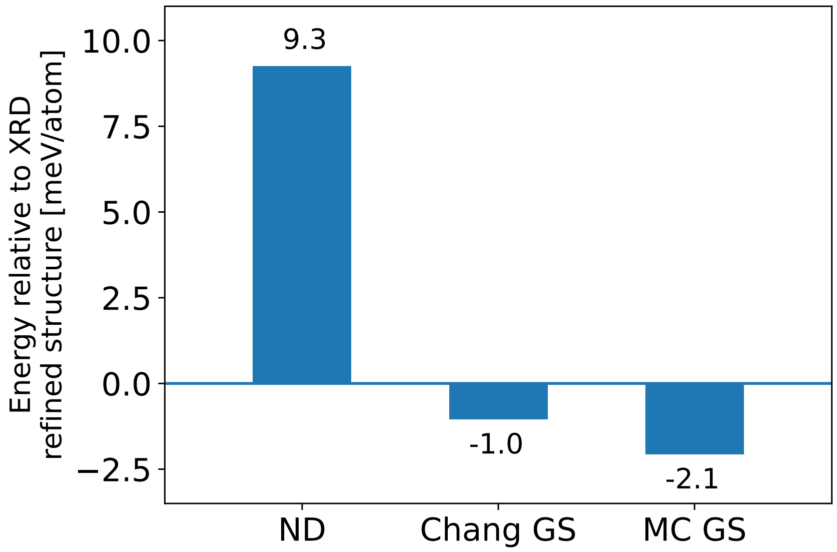

Figure S3: Energy of various low-energy $Li_7P_3S_{11}$ structures relative to the XRD refinement [meV/atom], calculated with r^2SCAN. Structures compared are the neutron diffraction refinement (ND), the ground state proposed by Chang and coworkers (Chang GS), and the ground state that we obtain from MC simulated annealing (MC GS).

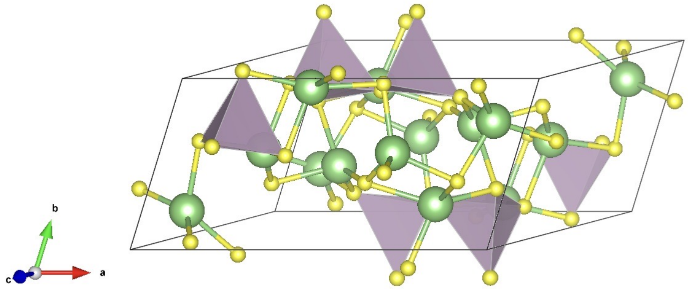

(a) XRD refinement.[2]

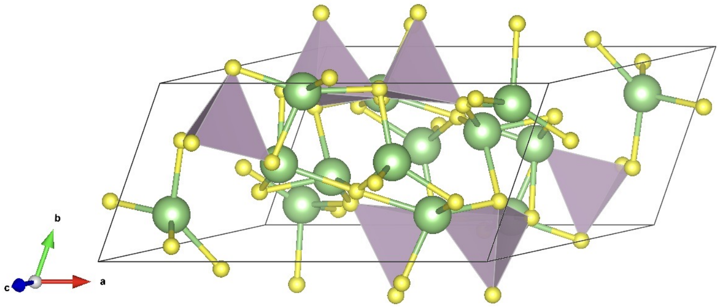

(b) ND refinement.[3]

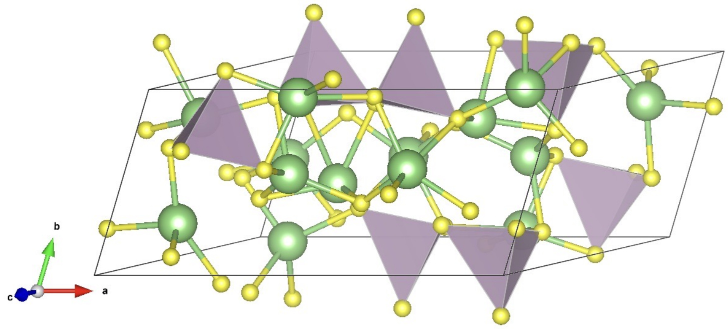

(c) Ground state proposed by Chang and coworkers.[4]

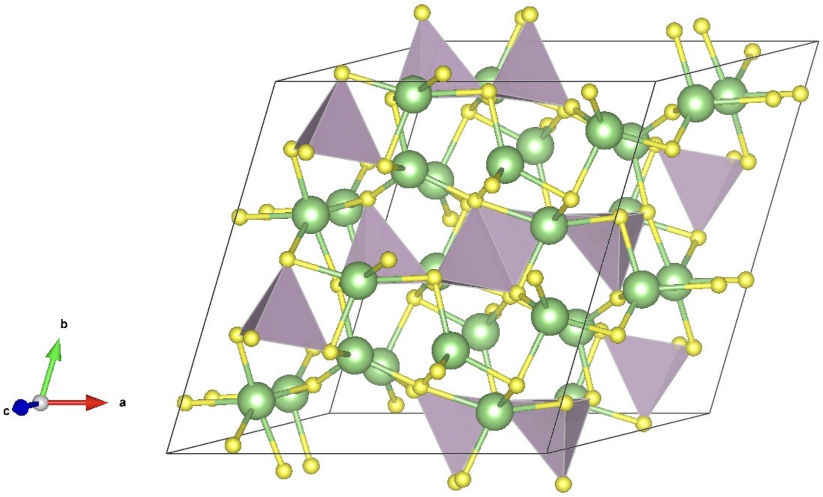

(d) Our proposed ground state (1x2x1 supercell) structure from MC simulated annealing.

Figure S4: Low energy orderings of $Li_7P_3S_{11}$ relaxed with r^2SCAN. Green – Li, yellow – S, purple tetrahedra – P.

IV: Cluster expansion details

TABLE SII: α-Li_3PS_4 primitive structure. Space group Cmcm (No. 63), (a, b, c) = (8.6435, 9.0462, 8.4779).[5]

Species	a	b	c	Wyckoff
Li1	0.724	0.353	0.528	16h
Li2	0.714	0	0	8e
Li3	0	0.196	0.25	4c
P	0	0.8306	0.25	4c
S1	0.3042	0.456	0.25	8g
S2	0	0.2949	0.5537	8f

TABLE SIII: β-Li_3PS_4 primitive structure. Space group Pnma (No. 62), (a, b, c) = (12.8483, 8.2772, 6.1512).[5]

Species	a	b	c	Wyckoff
Li1	0.151	0.532	0.896	8d
Li2	0.991	0.545	0.418	8d
Li3	0.084	0.75	0.196	4c
Li4	0.159	0.496	0.629	8d
P	0.912	0.75	0.823	4c
S1	0.845	0.548	0.702	8d
S2	0.064	0.75	0.746	4c
S3	0.895	0.75	0.154	4c

TABLE SIV: HT-Li_7PS_6 primitive structure. Space group F-43m (No. 216), a = 9.8482.[6]

Species	a	b	c	Wyckoff
Li1	0.6771	0.1771	0.4743	48h
Li2	0.953	0.047	0.246	48h
P	0.5	0	0	4c
S1	0.8794	0.1206	0.6206	16e
S2	0	0.5	0.5	4a
S3	0.25	0.25	0.75	4d

TABLE SV: $Li_7P_3S_{11}$ primitive structure. Space group P-1 (No. 2), (a, b, c) = (12.93, 6.13, 12.47), (α, β, γ) = (101.7, 115.0, 73.5).[4]

Species	a	b	c	Wyckoff
Li1	0.331	0.8678	0.908	2i
Li2	0.3619	0.598	0.1343	2i
Li3	0.784	0.47	0.26	2i
Li4	0.058	0.214	0.73	2i
Li5	0.641	0.4091	0.6632	2i
Li6	0.363	0.266	0.502	2i
Li7	0.861	0.706	0.866	2i
Li8	0.0	0.5	0.5	1e
Li9	0.93	0.0	0.84	2i
Li10	0.69	0.52	0.28	2i
Li11	0.99	0.79	0.64	2i
Li12	0.0	0.5	0.0	1d
Li13	0.96	0.7	0.37	2i
Li14	0.71	0.75	0.9	2i
Li15	0.8	0.69	0.24	2i
P1	0.2062	0.9666	0.5346	2i
P2	0.5071	0.962	0.7034	2i
P3	0.16	0.7302	0.9415	2i
S1	0.1394	0.7637	0.3805	2i
S2	0.1765	0.8948	0.6658	2i
S3	0.16	0.3171	0.532	2i
S4	0.3824	0.8403	0.5471	2i
S5	0.6694	0.7733	0.7116	2i
S6	0.4914	0.9205	0.8493	2i
S7	0.4906	0.2999	0.6926	2i
S8	0.1594	0.6698	0.0967	2i
S9	0.1721	0.0734	0.9538	2i
S10	0.3219	0.5126	0.936	2i
S11	0.0228	0.6491	0.8124	2i

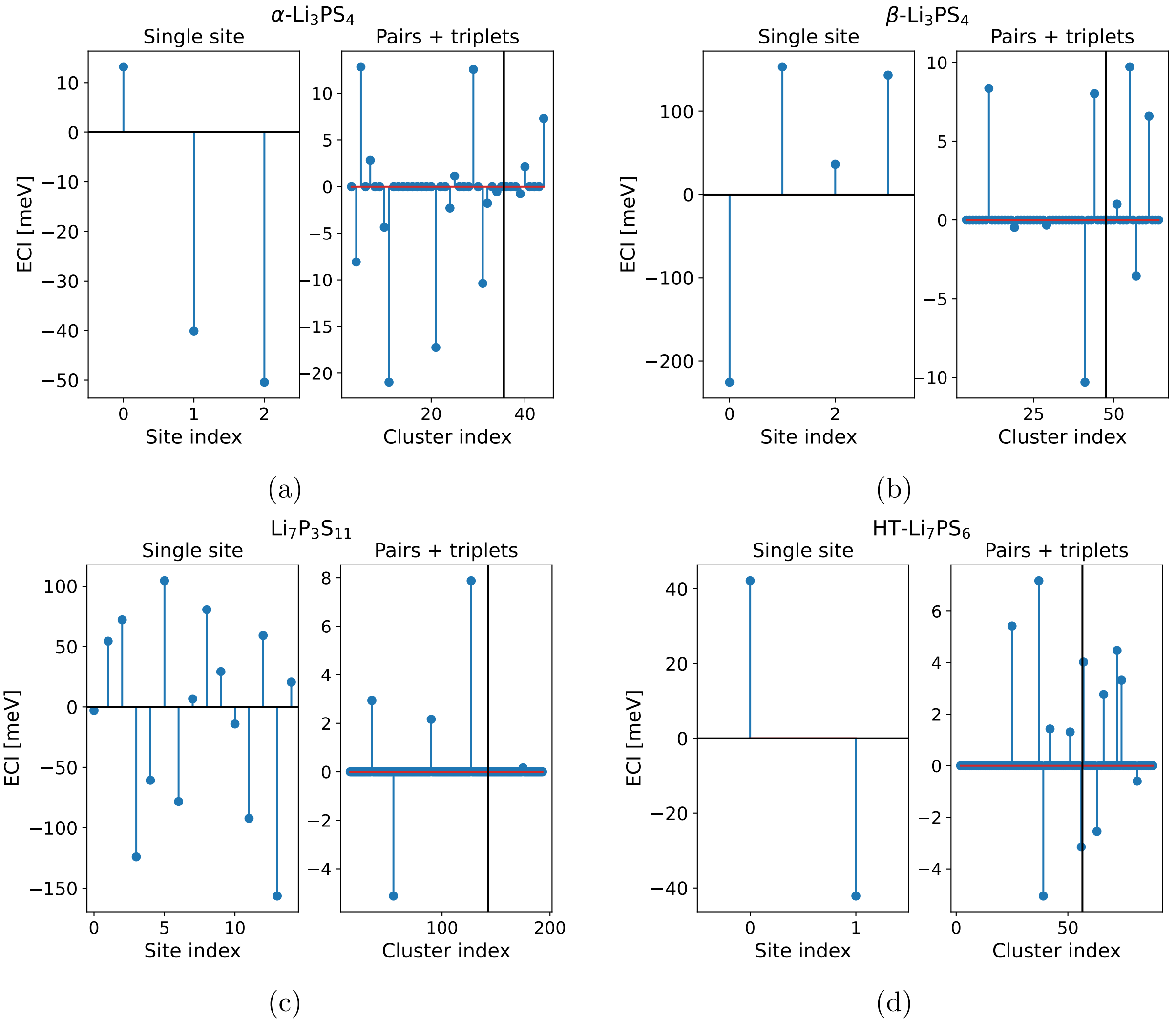

Figure S5: Effective cluster interactions (ECI) for each cluster expansion on the disordered superionic conductor phases. Vertical black line denotes division between pair and triplet clusters.

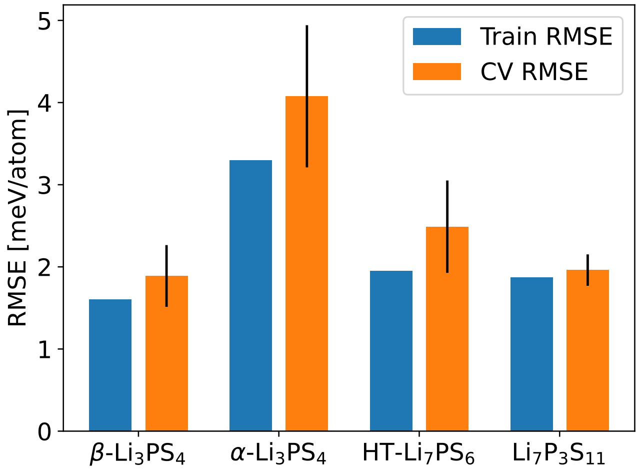

Figure S6: Cluster expansion training and cross-validation (CV) root-mean-squared errors (RMSE).

V: Formation energy and vibrational properties

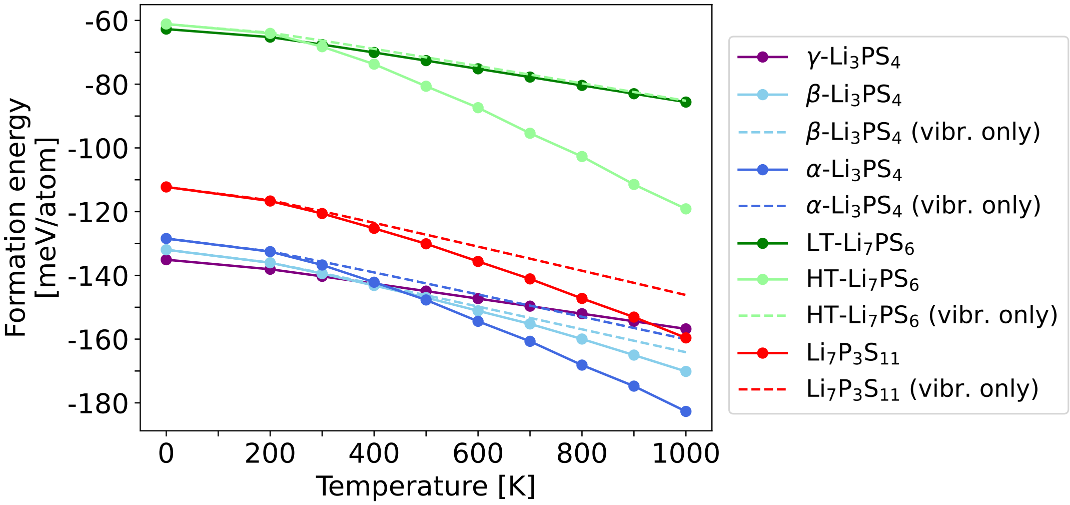

Figure S7: Formation free energies of all phases relative to the Li_2S and P_2S_5 end points as a function of temperature. Solid lines indicate free energy curves containing all entropy contributions and dashed lines have configurational entropy contributions removed.

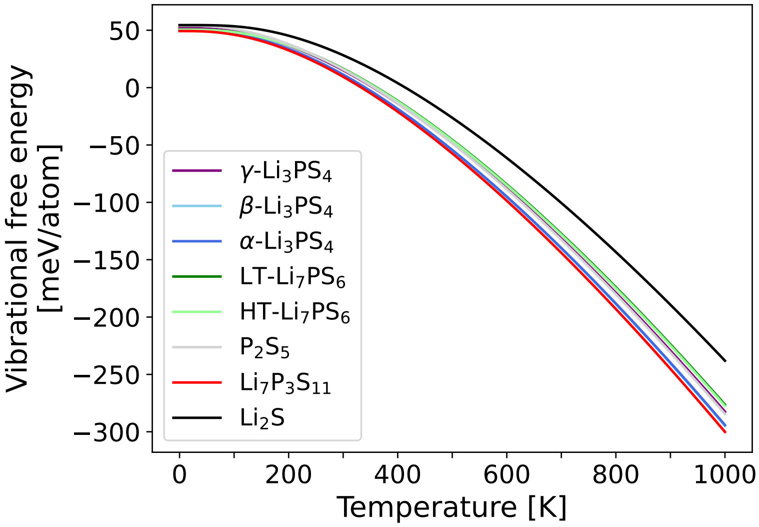

Figure S8: Vibrational free energy of each phase.

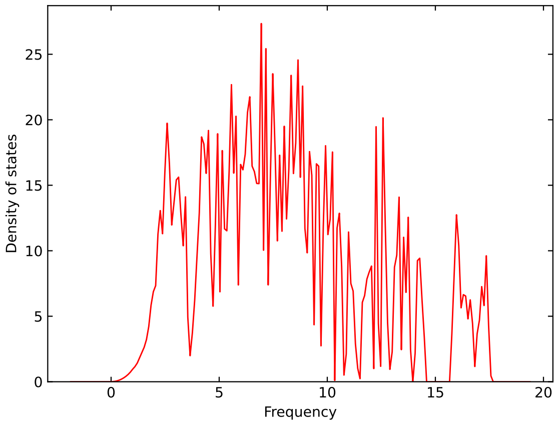

(a) LT-Li_7PS_6

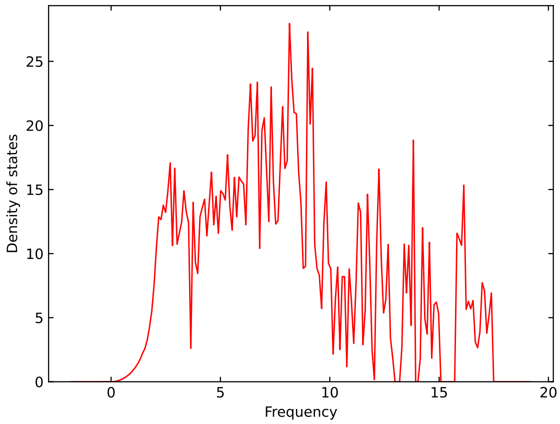

(b) HT-Li_7PS_6

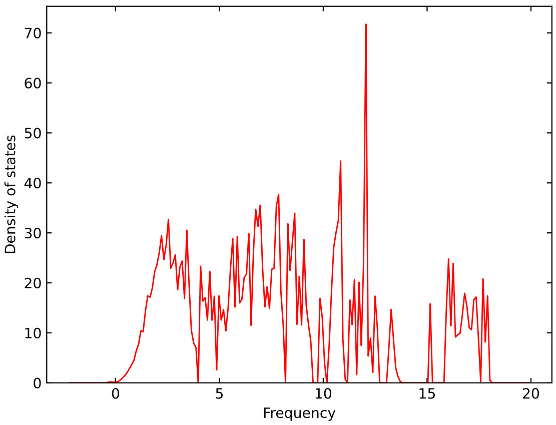

(c) $Li_7P_3S_{11}$

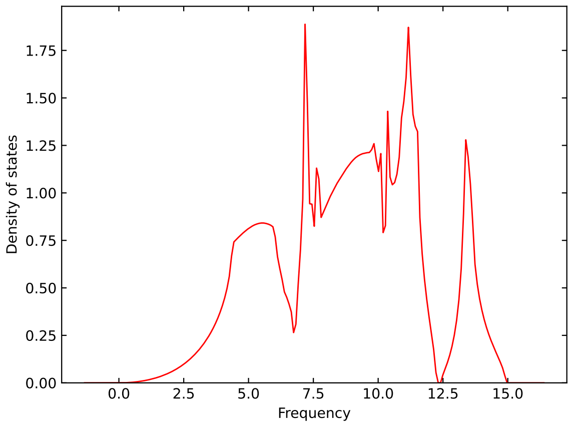

(d) Li_2S

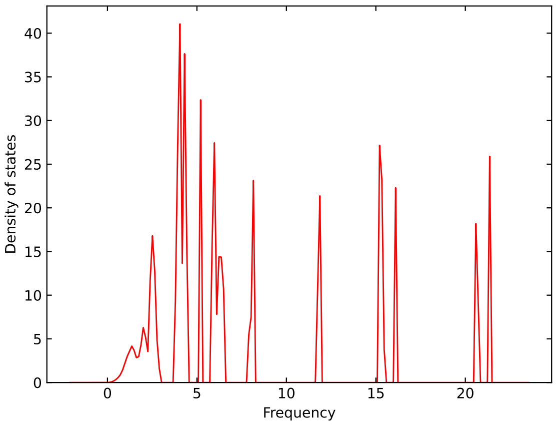

(e) P_2S_5

Figure S9: Phonon total density of states, normalized per primitive cell of the ground state.

References

(1) Kong, S.; Gün, ; Koch, B.; Deiseroth, H.; Eckert, H.; Reiner, C. Structural Characterisation of the Li Argyrodites Li7PS6 and Li7PSe6 and their Solid Solutions: Quantification of Site Preferences by MAS-NMR Spectroscopy. *Chemistry - A European Journal* **2010**, *16*, 5138–5147.

(2) Yamane, H.; Shibata, M.; Shimane, Y.; Junke, T.; Seino, Y.; Adams, S.; Minami, K.; Hayashi, A.; Tatsumisago, M. Crystal structure of a superionic conductor, Li7P3S11. *Solid State Ionics* **2007**, *178*, 1163–1167.

(3) Onodera, Y.; Mori, K.; Otomo, T.; Hannon, A. C.; Kohara, S.; Itoh, K.; Sugiyama, M.; Fukunaga, T. Crystal Structure of Li7P3S11 Studied by Neutron and Synchrotron X-ray Powder Diffraction. *Journal of the Physical Society of Japan* **2010**, *79*, 87–89.

(4) Chang, D.; Oh, K.; Kim, S. J.; Kang, K. Super-Ionic Conduction in Solid-State Li7P3S11-Type Sulfide Electrolytes. *Chemistry of Materials* **2018**, *30*, 8764–8770.

(5) Kaup, K.; Zhou, L.; Huq, A.; Nazar, L. F. Impact of the Li substructure on the diffusion pathways in alpha and beta Li3PS4: an in situ high temperature neutron diffraction study. *Journal of Materials Chemistry A* **2020**, *8*, 12446–12456.

(6) Schlenker, R.; Hansen, A.-L.; Senyshyn, A.; Zinkevich, T.; Knapp, M.; Hupfer, T.; Ehrenberg, H.; Indris, S. Structure and Diffusion Pathways in Li6PS5Cl Argyrodite from Neutron Diffraction, Pair-Distribution Function Analysis, and NMR. *Chemistry of Materials* **2020**, *32*, 8420–8430.
